# Unification of Treatments and Interventions for Tinnitus Patients (UNITI): a study protocol for a multi-center randomized clinical trial

**DOI:** 10.1186/s13063-021-05835-z

**Published:** 2021-12-04

**Authors:** Stefan Schoisswohl, Berthold Langguth, Martin Schecklmann, Alberto Bernal-Robledano, Benjamin Boecking, Christopher R. Cederroth, Dimitra Chalanouli, Rilana Cima, Sam Denys, Juliane Dettling-Papargyris, Alba Escalera-Balsera, Juan Manuel Espinosa-Sanchez, Alvaro Gallego-Martinez, Efi Giannopoulou, Leyre Hidalgo-Lopez, Michael Hummel, Dimitris Kikidis, Michael Koller, Jose A. Lopez-Escamez, Steven C. Marcrum, Nikolaos Markatos, Juan Martin-Lagos, Maria Martinez-Martinez, Marta Martinez-Martinez, Maria Mata Ferron, Birgit Mazurek, Nicolas Mueller-Locatelli, Patrick Neff, Kevin Oppel, Patricia Perez-Carpena, Paula Robles-Bolivar, Matthias Rose, Tabea Schiele, Axel Schiller, Jorge Simoes, Sabine Stark, Susanne Staudinger, Alexandra Stege, Nicolas Verhaert, Winfried Schlee

**Affiliations:** 1 Department of Psychiatry and Psychotherapy, University of Regensburg, Universitaetsstraße 84, 93053 Regensburg, Germany; 2 Department of Otolaryngology, Instituto de Investigacion Biosanitaria Ibs.GRANADA, Hospital Universitario Virgen de las Nieves, Granada, Spain; 3 Tinnitus Center, Charité- Universitätsmedizin Berlin, corporate member of Freie Universität Berlin und Humboldt Universität Berlin, Berlin, Germany; 4 Department of Physiology and Pharmacology, Karolinska Institutet, Stockholm, Sweden; 5 EXCELYA Hungary Kft., Budakalász, Hungary; 6 Department of Health Psychology, Katholieke Universiteit Leuven, Leuven, Belgium; 7 Department of Neurosciences, Research group Experimental Oto-Rhino-Laryngology, University of Leuven, Leuven, Belgium; 8 Department of Otorhinolaryngology - Head and Neck surgery, University Hospitals Leuven, Leuven, Belgium; 9 Multidisciplinary University Center for Speech-Language Pathology and Audiology, University Hospitals Leuven, Leuven, Belgium; 10 Terzo-Institute for Applied Hearing Research, ISMA, Sonneberg, Germany; 11 Otology & Neurotology Group CTS 495, Department of Genomic Medicine, GENYO, Center for Genomics and Oncological Research: Pfizer/University of Granada/Andalusian Regional Government, PTS Granada, Granada, Spain; 12 Department of Mental Health, Hospital Universitario Virgen de las Nieves, Granada, Spain; 13 Central Biobank Charité, Charité- Universitätsmedizin Berlin, corporate member of Freie Universität Berlin und Humboldt Universität Berlin, Berlin, Germany; 14 Department of Otolaryngology, Head and Neck Surgery, National and Kapodistrian University of Athens, Hippocrateion General Hospital, Athens, Greece; 15 Center for Clinical Studies, University Hospital Regensburg, Regensburg, Germany; 16 Department of Surgery, Division of Otolaryngology, Universidad de Granada, Granada, Spain; 17 Department of Otolaryngology, University Hospital Regensburg, Regensburg, Germany; 18 Department of Otolaryngology, Instituto de Investigacion Biosanitaria ibs. GRANADA, Hospital Universitario Clinico San Cecilio, Granada, Spain; 19 Center for Cognitive Neuroscience and Department of Psychology, University of Salzburg, Salzburg, Austria; 20 Department of Psychosomatic and Psychotherapy, Charité- Universitätsmedizin Berlin, corporate member of Freie Universität Berlin und Humboldt Universität Berlin, Berlin, Germany

**Keywords:** Tinnitus, Treatment, Hearing aids, Cognitive behavioral therapy, Sound therapy, Structured counseling, Multi-center, RCT

## Abstract

**Background:**

Tinnitus represents a relatively common condition in the global population accompanied by various comorbidities and severe burden in many cases. Nevertheless, there is currently no general treatment or cure, presumable due to the heterogeneity of tinnitus with its wide variety of etiologies and tinnitus phenotypes. Hence, most treatment studies merely demonstrated improvement in a subgroup of tinnitus patients. The majority of studies are characterized by small sample sizes, unstandardized treatments and assessments, or applications of interventions targeting only a single organ level. Combinatory treatment approaches, potentially targeting multiple systems as well as treatment personalization, might provide remedy and enhance treatment responses. The aim of the present study is to systematically examine established tinnitus therapies both alone and in combination in a large sample of tinnitus patients. Further, it wants to provide the basis for personalized treatment approaches by evaluating a specific decision support system developed as part of an EU-funded collaborative project (Unification of treatments and interventions for tinnitus patients; UNITI project).

**Methods/study design:**

This is a multi-center parallel-arm randomized clinical trial conducted at five different clinical sites over the EU. The effect of four different tinnitus therapy approaches (sound therapy, structured counseling, hearing aids, cognitive behavioral therapy) applied over a time period of 12 weeks as a single or rather a combinatory treatment in a total number of 500 chronic tinnitus patients will be investigated. Assessments and interventions are harmonized over the involved clinical sites. The primary outcome measure focuses on the domain tinnitus distress assessed via the Tinnitus Handicap Inventory.

**Discussion:**

Results and conclusions from the current study might not only provide an essential contribution to combinatory and personalized treatment approaches in tinnitus but could also provide more profound insights in the heterogeneity of tinnitus, representing an important step towards a cure for tinnitus.

**Trial registration:**

ClinicalTrials.gov NCT04663828. Registered on 11 December 2020.

**Supplementary Information:**

The online version contains supplementary material available at 10.1186/s13063-021-05835-z.

## Background

Following a recent multidisciplinary consensus, tinnitus is termed as “the conscious awareness of a tonal or composite noise for which there is no identifiable corresponding external acoustic source” [1] whereby a continuous perception over 6 months constitutes a chronification [2]. Frequent causes for the emergence of tinnitus range from noise trauma, presbyacusis to intake of ototoxic medication, potentially provoking pathological neural alterations in the central auditory pathway [3]. Approximately 10–15% of the global adult population are affected by this phantom sound perception [4], whereas 2–3% are particularly suffering from tinnitus [5, 6]. In many cases, tinnitus generates a high level of suffering and can be accompanied by various comorbidities such as depression, anxiety, or sleep disorders [7–10], explicitly defined as tinnitus disorder [1]. Currently, there is no general treatment respectively a cure for tinnitus existent. Available treatment approaches cover a broad spectrum of interventions from pharmacology [6], neurostimulation [11], cochlear implants [12] to different sound therapies [13, 14] or hearing aids [15, 16]. While the European guidelines for tinnitus [17] give a weak recommendation for the application of amplification devices in tinnitus patients with hearing loss, there is currently no explicit recommendation for pharmacological interventions or neurostimulation [17]. Up to now, cognitive behavioral therapy approaches exhibit the best body of evidence for the treatment of tinnitus [18, 19] with a strong recommendation according to European guidelines [17]. Even though tinnitus research has been impressively expanded over the past decade, the majority of studies suffers from methodological shortcomings such as heterogeneous patient samples, imprecisely defined therapeutic interventions, relatively small sample sizes, and a lack of predefined primary outcomes and data analysis strategies [20, 21]. Beyond tackling these limitations in prospective studies, interdisciplinary multi-center randomized clinical trials (RCT) could help to further increase the validity and interpretability of results.

The complexity of tinnitus with a wide variety of phenotypes and different etiologies plus the uncertainty about underlying pathophysiological processes make the quest in finding an appropriate treatment rather difficult [3, 22]. In most of the studies, only a subgroup of patients exhibits improvement to a certain intervention ranging from 1% to more than 35%. However, currently none of the treatment approaches has sound and universal findings [23]. Thus a common treatment for all tinnitus subtypes is most unlikely, highlighting the necessity of so-called precision medicine or rather personalized treatment concepts in tinnitus [24, 25]. A potential proceeding would be the identification of demographic or tinnitus-related characteristics, potentially capable to predict a patient’s response to a certain type of intervention [26]. Such predictive markers could facilitate a so-called Decision Support System (DSS), which could assist clinicians in selecting the most promising tinnitus treatment on an individual patient level [20].

Currently available tinnitus treatments mostly aim at different systems and domains, e.g., the auditory system (AS) or the central nervous system (CNS) respectively focuses mainly on a single target of the underlying pathophysiology. At best, tinnitus treatments should integrate all involved components/ systems [27]; hence, a combination of different therapeutic approaches could provide further remedy. Studies focusing on combinatory interventions range from combinations of hearing aids with sound generators [28–30], simultaneous sound and somatosensory stimulations [31, 32], application of counseling together with tinnitus masking termed tinnitus retraining therapy [13, 22, 33, 34], to brain stimulation in combination with relaxation [35] as well as multimodal therapies [36, 37], though past studies are not capable to provide a clear superiority of combined interventions. A systematic examination of several different single and combinational interventions is currently not available, highlighting the need for more profound investigations in this regard.

The present study protocol describes the methodological procedure of the UNITI-RCT (Unification of Treatments and Interventions for Tinnitus Patients – Randomized Clinical Trial), which constitutes the centerpiece of the EU-funded UNITI project. The primary objective of the UNITI project is the development of a computational model to predict patients’ responses to distinct treatments in order to facilitate personalized therapies in tinnitus. For a detailed overview of goals and procedures of the UNITI project, see Schlee et al. [38].

### Objectives

The attempt of the UNITI-RCT is to not only overcome the shortcomings of previous studies, but also pave the way for personalized medicine approaches in tinnitus. For this purpose, a multi-center parallel-arm superiority RCT, implemented and harmonized among five clinical sites across the EU, combining and investigating selected existing therapies evaluated in the European guidelines for tinnitus [17], is conducted. The main objective of the UNITI-RCT is (1) to scrutinize if a combinational therapy is more effective than a single therapy for the treatment of chronic tinnitus. Additionally, (2) the outcomes of each utilized intervention will be compared against each other; (3) several treatment groups will be formed and analyzed based on whether participants received a certain treatment type alone or in combination with another treatment, (4) whether participants received a certain type of intervention at all (either alone or in combination), and (5) whether the received interventions targeted on one or two organ levels—the ear or the CNS (ear- vs. brain-mediated interventions). Moreover, (6) the development of a specific DSS, which will be based on demographic, psychological, audiological, electrophysiological, and genetic parameters, for patient-specific data-driven treatment suggestions [39] will be validated over the course of the UNITI-RCT.

## Methods

For the reporting of the methodological approach of the UNITI-RCT, the SPIRIT guidelines have been used [40].

### Study design

The study is designed as a multi-center RCT which investigates the effect of four different tinnitus therapy approaches applied as single or combinatory treatments over a time period of 12 weeks in 500 patients with mild to severe tinnitus distress. The clinical trial has been registered at ClinicalTrials.gov (NCT04663828; trial registration dataset in the supplemental material) and will be completed in five different clinical sites across the European Union.

1. University of Regensburg, Regensburg, Germany (RCT coordinator)
2. Charité – Universitaetsmedizin Berlin, Berlin, Germany
3. Ethniko Kai Kapodistriako Panepistimo Athinon, Athens, Greece
4. Hospital Universitario Virgen de las Nieves/Hospital Clinico Universitario San Cecilio, Granada, Spain
5. Katholieke Universiteit Leuven, Leuven, Belgium

### Sample size determination/ effect size calculation

Since there is no reliable data for the main purpose of the study (single vs. combinational treatment) available from which an effect size for sample calculation can be deduced, literature from a stepped-care approach involving counseling, sound therapy, and CBT was used for this purpose [18]. This study exhibited an effect size of 0.52 after 8 months when a stepped-care approach including a combination of treatments was contrasted against treatment as usual. This approach is not fully comparable to the current RCT (single vs. combinational treatment), as also treatments with much lower effect sizes are included in the current study (hearing aids, sound therapy, structured counseling), and effect size was estimated conservatively as about 0.26. With a significance level of 5% and a power of 80% (two-sided test), the necessary sample size is 468. Taking also drop-outs into account, the aim is to investigate a total sample size of *N* = 500.

### Study population

As already mentioned above a total number of 500 participants will be investigated in the course of this RCT. Each of the five clinical sites has the objective to examine *n* = 100 patients with chronic subjective tinnitus for the RCT with respect to specific inclusion and exclusion criteria. Potential candidates will be recruited via media advertising (according to local regulations) as well as on an individual basis at the clinical sites through, e.g., information sheets, word of mouth, or in conversations with medical staff. In order to be eligible for participation, potential participants have to meet the following criteria as outlined in Table 1.

**Table 1:** Eligibility criteria

Inclusion criteria	Exclusion criteria
- Primary complaint tinnitus - Chronic tinnitus (≥ 6 months) - Age between 18 and 80 years - A score of ≥ 18 in the Tinnitus Handicap Inventory (THI; [41] - at least mild tinnitus distress - A score of > 22 in the Montreal Cognitive Assessment (MoCa; [52])-absence of mild cognitive impairment - Ability and willingness to use the UNITI mobile applications on smartphones - Openness to use a HA (if indication and allocation to HA group) - Ability to understand and consent to the research (hearing ability, intellectual capacity) - Ability to participate in all relevant visits (no plans for, e.g., long-term holidays or pregnancy^a^) - Existing drug therapies with psychoactive substances (e.g., antidepressants or anticonvulsants) must be stable for at least 30 days at the beginning of the therapeutic intervention. The drug therapy should remain constant during the course of the study. Necessary changes do not constitute an exclusion criterion per se, but need to be recorded.	- Objective tinnitus or heartbeat-synchronous tinnitus as primary complaint - Otosclerosis/ acoustic neuroma or other relevant ear disorders with fluctuation hearing - Present acute infections (acute otitis media, otitis externa, acute sinusitis) - Meniere’s disease or similar syndromes (but not vestibular migraine) - Serious internal, neurological or psychiatric conditions - Epilepsy or other disorders of the central nervous system (e.g., brain tumor or encephalitis) - Clinically relevant drug, medication or alcohol abuse up to 12 weeks before study start - Severe hearing loss-inability to communicate properly in the course of the study - One deaf ear - Missing written informed consent - Start of any other tinnitus-related treatments, especially hearing aids, structured counseling, sound therapy (with special devices; expecting long-term effects) or cognitive behavioral therapy in the last 3 months before the start of the study^b^

^a^ Due to specific standards of the local ethics committee at the clinical site in Granada, Spain , with respect to the conduction of RCTs, all female participants will be tested with regard to an existing pregnancy

^b^ If a HA has already been worn 3 months before screening, eligible candidates are allowed to participate, but are automatically assigned to the group with no HA indication

### Outcome measures and assessments

All measures, assessments, and documentations are harmonized among the clinical sites. Besides the below listed standardized measures and assessments, participants’ comorbidities as well as concomitant treatments and medications will be recorded. All types of medication (even over-the-counter drugs), which have been ingested within the last 3 months before screening respectively up to the time of study starting, and which will be ingested during the study are documented with respect to dose, administration, and begin and stop date. Further, all types of treatments which have been performed within the last 3 months before screening respectively up to the time of study starting and/or will be performed during the course of the trial are documented with respect to frequency and begin/ stop date.

#### Primary outcome

The primary outcome of the current RCT is focused on the domain tinnitus distress. Changes in tinnitus distress with respect to the applied interventions will be assessed via the total score of the Tinnitus Handicap Inventory (THI) [41].

#### Secondary outcome

Several other standardized tinnitus- and health-related questionnaires will be used as secondary outcomes in the course of this RCT:

1. Tinnitus Functional Index (TFI) [42]
2. Mini Tinnitus Questionnaire (Mini-TQ) [43]
3. Tinnitus numeric rating scales (NRS) [44]
4. World Health Organization – Quality of Life abbreviated (WHOQoL-Bref; https://www.who.int/healthinfo/survey/WHOQOL_BREF.pdf?ua=1)
5. Clinical Global Impression Scale - Improvement (CGI-I) [45]
6. Patient Health Questionnaire for Depression (PHQ-D/PHQ-9) [46, 47]

#### Sample description and other measures

1. European School of Interdisciplinary Tinnitus Research Screening Questionnaire (ESIT-SQ) [48]
2. Tinnitus Sample Case History Questionnaire (TSCHQ) [49]
3. Questionnaire on Hypersensitivity to Sound (GUF) [50]
4. Big Five Inventory-2 (BFI-2) [51]
5. Montreal Cognitive Assessment (MoCA) [52]
6. Social Isolation Electronic Survey (SOISES) [53]—a subset of 11 questions sensitive for tinnitus distress change will be used, further designated as Mini-SOISES
7. Attitudes Towards Amplification Questionnaire (ATAQ)—a subset of questions (baseline, 8; end of treatment, 7) associated with hearing aids taken from the Attitudes towards Loss of Hearing Questionnaire (ALHQ) [54]
8. Fear of Tinnitus Questionnaire (FTQ) [55]

#### Electrophysiological measures

Two types of electrophysiological measures are performed in the course of this RCT—auditory brain stem responses (ABR) and auditory middle latency responses (AMLR). ABRs represent the synchronized neural activity along the auditory pathway evoked by a serial presentation of acoustic stimuli (e.g., clicks). ABR is considered to be a robust electrophysiological method to assess the functional integrity of the auditory pathways. Normally, up to five waves occur in the ABR response. The wave I of the ABR reflects activity of the spiral ganglion cells at the distal part of the eighth auditory nerve, the wave II from the globular cells in the cochlear nucleus, the wave III is generated by the cochlear nucleus spherical cells and globular cells, and wave IV and wave V are generated from the medial superior olive and its projections to the nuclei in the lateral lemniscus and the inferior colliculus [56]. These electrophysiological responses are typically less than a microvolt in amplitude [57, 58]. AMLRs are typically composed of a set of positive (P-waves) and negative (N-waves) waves. AMLRs are sensitive potentials for the processing of low-frequency tones. The difference found between the behavioral auditory threshold and the electrophysiological threshold is of approximately 10 dB [59]. Further, they may be used for the investigation of functional integrity in the auditory pathway or the assessment of non-organic hearing loss. Both measures will be recorded with four external electrodes placed over the forehead and behind the ear during the presentation of standardized acoustic signals while patients are lying in a quiet room. Measurements will be performed by trained medical and/ or study staff. These measurements will not be used as outcome measures, but will be analyzed as potential prognostic factors for treatment response.

#### Audiometric and tinnitometric measures

Otological examinations will be executed according to state-of-the-art medical practice. Audiological and/ or tinnitometric assessments will be conducted by responsible medical and/ or trained study staff with a standard clinical audiometer. Pure tone air-conduction and bone-conduction audiometry will be recorded according to the guidelines from the British Society of Audiology - http://www.thebsa.org.uk/wp-content/uploads/2014/04/BSA_RP_PTA_FINAL_24Sept11_MinorAmend06Feb12.pdf. Individual tinnitus characteristics such as pitch and loudness will be determined via the presentation of different frequencies (or narrow band noise in the case of noise-like tinnitus). Participants have to decide which stimulus is closer to their tinnitus percept in a forced choice paradigm. Two different pure tones with a difference of two octaves (1 kHz and 4 kHz) will be consecutively presented and patients have to select the frequency which is closer to their tinnitus pitch (not intensity). Next, the selected frequency plus a different frequency closer to the one selected (one octave difference) will be presented and patients have to make the same decision again. The procedure goes on, keeping the closest frequency for the next round, until patients confirm the same frequency three times. In each round, different neighboring frequencies are presented. Frequencies used include 250, 500, 1000, 2000, 3000, 4000, 6000, and 8000 Hz, presented at 20 dB above the individual hearing level of the respective frequency.

Subsequently, tinnitus loudness is determined by the comparison of the determined tinnitus frequency at different loudness levels starting from the individual hearing level (5 dB steps).

The procedures are conducted in one ear in case of unilateral (contralateral to tinnitus percept) or symmetric bilateral tinnitus and in two ears in cases of asymmetric tinnitus.

In order to assess patients’ minimum masking level, a narrow band noise centered at the previously defined tinnitus frequency is presented in an ascending way (5 dB steps, starting from individuals hearing threshold) ipsilateral to the tinnitus percept until an adequate level to cover the patients’ tinnitus is reached.

The phenomenon of a brief tinnitus percept suppression following acoustic stimulation is termed residual inhibition [60] and will be evaluated via 30-s and 2-min presentations of a pure tone and a broad band noise in accordance with the determined individual tinnitus frequency.

#### Blood samples

Blood samples are taken once before treatment start on a voluntary basis and are therefore not mandatory for trial participation. Participants are informed about blood sample collection and how their samples will be handled in detail (with respect to EU and country-specific regulations). The blood samples are sent to the Center for Genomics and Oncological Research (GENyO), Universidad de Granada (Granada, Spain) and the plasma samples to the Karolinska Institutet (Stockholm, Sweden) for further analysis of genetic parameters and plasma proteins respectively. Both laboratories are part of the UNITI consortium [38]. Storage, shipping, and analysis of participants biological samples are done according to the EU GDPR (2016/679) as well as country-specific legislations.

Peripheral blood samples from patients included in the clinical trial will be obtained in EDTA coated tubes. After centrifugation at 1500*g* during 10 min, plasma and cellular fractions will be separated. Plasma will be immediately frozen at − 80° to prevent protein degradation.

##### DNA isolation and genome sequencing

DNA will be isolated from the buffy coat by the QIAamp DNA blood Mini kit (QIAGEN), according to the manufacturer’s protocol. DNA quality will be assessed by absorbance measurements (Nanodrop 2000c, Thermo Scientific) and by 260/280 and 230/260 indexes to determine protein and salt content. DNA concentration will be determined by a fluorimetric method (Qubit) and Quant-iT technology (Invitrogen) and DNA integrity will be evaluated in 1% agarose gels and by Bioanalyzer technology [61].

For whole-genome sequencing, DNA libraries will be prepared by using the Truseq DNA PCR-free kit (350 bp). DNA will be sequenced in an Ilumina NovaSeq 6000 sequencing platform 150 paired-end reads (150 × 2 bp) with a throughput 30× (90 Gb/sample). Bioinformatic analysis will include alignment and mapping of raw data to the latest updated reference genome (GRCh38.p13), preprocessing of FASTQ and generation of BAM files, variant calling to obtain VCF files following GATK Best Practices guidelines, and annotation using Ensemble Variant Effect Predictor.

Analyzing the whole genome using next-generation sequencing delivers a base-by-base view of all genomic alterations, including single-nucleotide variants (SNV), small insertions and deletions, and large structural variations (copy number changes). Paired-end whole-genome sequencing involves sequencing both ends of a DNA fragment, which increases the likelihood of alignment to the reference and facilitates detection of genomic rearrangements, repetitive sequences, and gene fusions. Gene burden tests will be used to compare burden of loss of function SNV and structural variants in synaptic genes in patients with tinnitus, using Non-Finnish European dataset from gnomAD v3 as population database to estimate allelic frequencies [62].

##### Protein analyses

Protein biomarkers for patients included in the RCT will be initially assessed by a proximity extension assay (PEA) from O’LINK. Control groups will include subjects with and without hearing loss. PEA multiplex analysis in 500 tinnitus cases and 2728 non tinnitus control blood samples from the STOP study (https://stop.ki.se/) will be performed. An immune response biomarker panel directed against 96 proteins implicated in inflammatory diseases will be used, since several studies have found inflammation to be involved in various forms of hearing loss. Therefore, inflammatory biomarkers could potentially be associated with tinnitus. Standard biostatistical analyses will be performed, including basic descriptive and univariate statistics as well as receiver operating characteristics curves.

Genetic and blood biomarkers will serve as a basis for determining tinnitus subtypes and evaluating persons’ treatment response at the clinical sites through the procedure.

### Treatment conditions

Four different types of interventions will be included in the present RCT. Two of them mainly target on the auditory aspects of tinnitus (sound therapy; hearing aid), whereas the other two especially target the CNS (structured counseling; cognitive behavioral therapy). Each intervention will be harmonized among the participating clinical sites in view of procedure, technical equipment, and trained research staff. This will be achieved via specific Standard Operation Procedure documents and a detailed step-by-step description of procedures as well as the conduction of dedicated workshops from leading experts in the particular field for each intervention. Hereinafter, the used interventions are described.

#### Sound therapy (ST)

Sound therapy will be self-administered on a daily basis and conducted via a dedicated mobile application for Android and iOS in accordance to the European regulations for medical software (IEC 62304, IEC 82304). This mobile application will contain a set of 64 different stimuli comprised of various artificial and naturalistic sounds and an application of different state-of-the-art filter or modulation techniques, e.g., amplitude modulated tones or noises [63, 64] as well as filtered music [65]. Intensity and length of sound stimulation can be adjusted by the user. Maximum loudness for all types of sound stimulation will be 85 dB. Patients’ behavioral data (tinnitus loudness after stimulation) as well as type of sound, loudness, and play duration/ repetitions will be collected for each conducted stimulation. Further, the time of app usage will be recorded. All personal data will be treated with the upmost confidentiality and multiple efforts will be made in order to pseudo-anonymize the collected data and to protect participants’ identities. EU General Data Protection Regulation (GDPR, 2016/679) will apply for the mobile application, which are outlined in the data management plan in the supplemental material.

#### Hearing aid (HA)

Participants will be stratified according to their hearing function. Patients with a hearing aid indication will represent one strata and only these patients will be randomized for the option “hearing aid therapy.” Conventional commercially available hearing instruments will be utilized with all tinnitus-specific sound interventions deactivated, in order to disentangle the effect of amplification from the effects of sound therapy. Specifically, all participants were fitted bilaterally with mini-behind-the-ear hearing instruments (Type Signia Pure 312 7X; Sivantos Pte. Ltd., Singapora, Republic of Singapore/ WSAudiology, Lynge, Denmark), with acoustic coupling achieved via non-custom, non-occluding eartips. Gain assignments appropriate for each individual’s hearing loss will be applied according to the National Acoustic Laboratories-Non-Linear 2 generic amplification prescription procedure [66] and verified using probe microphone measures. As it has been reported that numerous signal processing characteristics might significantly affect the impact of amplification on tinnitus, all signal processing features, excepting only acoustic feedback reduction and impulse noise reduction algorithms, will be deactivated during the 12-week hearing aid trial. Verification of hearing aid gain assignments and signal processing will be performed by audiologists, hearing aid acousticians, or trained study personnel, in accordance with the appropriate practice guideline of the British Society of Audiology (http://www.thebsa.org.uk/wpcontent/uploads/2018/05/REMS-2018.pdf). Participants will be encouraged to use their hearing aids for at least 10 h per day and will be allowed to keep their hearing devices following successful completion of the RCT.

#### Structured counseling (SC)

A standardized protocol developed from a team of psychologists will provide structured patient education, counseling, and tips for a life with tinnitus on a daily basis. The protocol is oriented towards recent European Tinnitus Guidelines [17] and comprised of 12 chapters:

1. Basic facts about tinnitus
2. Beginner facts about tinnitus
3. Beginner facts about the brain and sound perception
4. Important vocabulary
5. Myths and misconceptions about tinnitus
6. Diagnosis of tinnitus
7. Special types of tinnitus
8. Therapeutic approaches
9. Psychology of tinnitus
10. My behavior and my tinnitus
11. The science on tinnitus
12. Switch perspective: How would you advice others?

Structured counseling will be equally implemented via a mobile application on Android and iOS in compliance with the European regulations for medical software (IEC 62304, IEC 82304)—details can be found in the data management plan in the supplemental material. At the end of each chapter, there will be a short quiz to repeat and consolidate the content. Answers and scores of the quizzes as well as time of app usage will be collected according to the General Data Protection Regulation (GDPR, 2016/679). Patients will be encouraged to use the specifically developed app for structured counseling. However, alternatively the information contained in the app can also be provided in printed form.

#### Cognitive behavioral therapy for tinnitus (CBT4T)

A specially designed cognitive behavioral therapy (CBT) program for tinnitus patients will be applied by psychologist or psychotherapists by training. CBT4T will be performed in weekly face-to-face group meetings á 1.5-2 h over the 12-week treatment phase. One face-to-face screening visit will take place before the group session. The maximum number of participants per group will be six tinnitus patients. Psychologists and psychotherapists will carry out the CBT4T and are trained by workshops before the treatment. The theoretical concept and the content of the treatment of CBT4T is based on the concept of exposure therapy in anxiety disorders or chronic pain (cognitive restructuring via exposition to fear expectations) and a fear-avoidance model [18, 67, 68]. Short psychoeducation at the beginning of the treatment is followed by exposure exercises in group sessions.

### Trial procedure

The UNITI-RCT will be conducted at the five clinical sites as well as in patients’ homes. All patients have to make at least four visits in the respective clinical sites over a period of at least 10 months consisting of screening, baseline visit, interim assessment, final visit at treatment end, and a follow-up visit 36 weeks after the baseline assessment. Patients who will be randomly assigned to CBT4T have to additionally visit the respective clinical site twelve times in order to participate in the CBT sessions. All other treatments are conducted without local restrictions.

Screening, baseline, and treatment start can be performed on the same day. In this case, relevant assessments are only performed once. Furthermore, an additional visit in the screening period can be added for purposes of screening/ baseline procedures. Prior to the first screening visit, there will be an online pre-screening in order to preselect potentially appropriate participants.

The interval between baseline and treatment start should be maximally 4 weeks. If the interval is longer, an additional visit will be done (without ESIT-SQ, TSCHQ, BFI-2, ATAQ, electrophysiological measurements, or otological examination). Blood samples will be taken before treatment start. Consent for the participation at the genetic and plasma protein tests is independent from the consent to participation in the clinical trial. In case of a combinational treatment, the second treatment will start maximum ten working days after the first (e.g., HA and CBT4T). Moreover, a facultative additional follow-up assessment 48 weeks after baseline measurements is planned. If a patient drops out for any reason or wishes to discontinue the treatment prematurely, there is the possibility to continue with study visit participation.

**Fig. 1:**
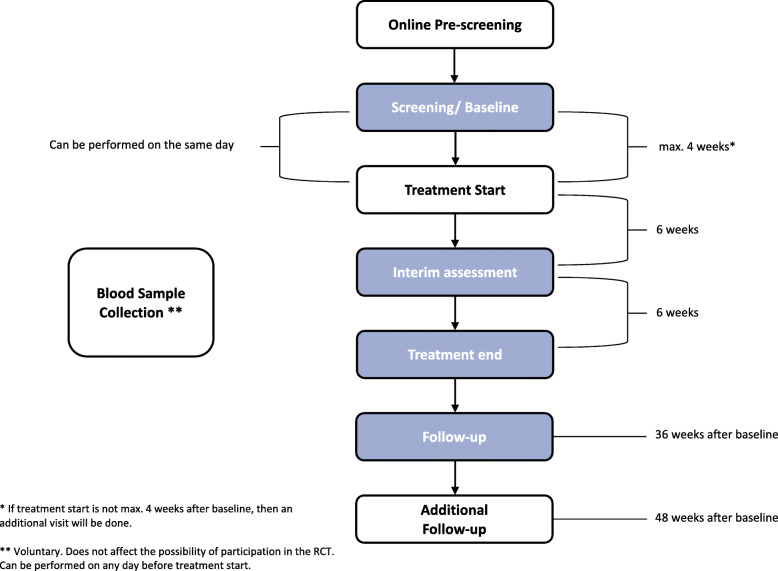
Study procedure. An overview of all study visits/ measurement points plus the corresponding time periods between the visits is illustrated. For each study visit, a deviation of ± 7 days is allowed. In order to preselect potentially appropriate participants, an online pre-screening precedes the actual screening. Screening and baseline can be performed on the same day. Baseline has to be done at maximum 4 weeks before treatment start; otherwise, there will be an extraordinary visit. An additional follow-up visit 48 weeks after baseline can be conducted on a voluntary basis. Blood samples can be taken at any time point before treatment start (after informed consent) and is not mandatory for participation in the UNITI-RCT

For each study visit, a deviation of ± 7 days is permitted. Figure 1 gives an overview of the study procedure, while in Table 2 the assessments for all measurement points are outlined. In the following sections, each visit is shortly described in a chronological order.

**Table 2:** Schedule of assessments

	Pre-screening	Screening	Baseline	Treatment start	Interim visit	Final visit = end of treatment	Follow-up	Additional follow-up
**ICF**	**A**^**a**^	**A**						
**Eligibility criteria**	**A**	**A**	**A**					
**ESIT-SQ**			**A**					
**TSCHQ**			**B**					
**Mini TQ**	**A**	**A**	**A**		**A**	**A**	**A**	**B**
**Tinnitus numeric rating scales**		**A**	**A**		**A**	**A**	**A**	**B**
**TFI**		**A**	**A**		**A**	**A**	**A**	**B**
**THI**	**A**	**A**	**A**		**A**	**A**	**A**	**B**
**WhoQol-BREF**		**A**	**A**		**A**	**A**	**A**	**B**
**BFI-2**			**A**					
**CGI-I**					**A**	**A**	**A**	**B**
**GUF**		**B**	**B**		**B**	**B**	**B**	**B**
**PHQ-D**	**A**	**A**	**A**		**A**	**A**	**A**	**B**
**Mini-SOISES**			**A**		**A**	**A**	**A**	**B**
**ATAQ**			**B**^**b**^			**B**^**b**^		
**FTQ**			**B**		**B**	**B**	**B**	**B**
**MoCA**		**A**						
**Randomization**			**A**					
**Blood sampling**			**B**^**c**^					
**Otological examination**		**A**				**A**	**B**	**B**
**Audiometry**		**A**				**A**	**B**	**B**
**Loudness match**		**A**				**A**	**B**	**B**
**Pitch match**		**A**				**A**	**B**	**B**
**Maskability**		**A**				**A**	**B**	**B**
**Residual inhibition**		**A**				**B**	**B**	**B**
**ABR**			**A**				**B**	**B**
**AMLR**			**A**				**B**	**B**
**Treatment**				**A**	**A**	**A**		
**Comorbidities**		**A**	**A**	**A**	**A**	**A**	**A**	**B**
**Concomitant medication/ treatment**		**A**	**A**	**A**	**A**	**A**	**A**	**B**
**Adverse events**					**A**	**A**	**A**	**B**

*A* mandatory; *B* voluntary; *ICF* Informed Consent Form; *ESIT-SQ* European School of Interdisciplinary Tinnitus Research Screening Questionnaire; *TSCHQ* Tinnitus Sample Case History; *Mini-TQ* Mini Tinnitus Questionnaire; *TFI* Tinnitus Functional Index; *THI* Tinnitus Handicap Inventory; *WhoQol-BREF* World Health Organization Quality of Life – abbreviated; *BFI-2* Big Five Inventory-2; *CGI-I* Clinical Global Impression Scale – Improvement; *GUF* Questionnaire on Hypersensitivity to Sound; *PHQ-D* Patient Health Questionnaire for Depression; *SOISES* Social Isolation Electronic Survey; *ATAQ* Attitudes Towards Amplification Questionnaire; *FTQ* Fear of Tinnitus Questionnaire; *MoCA* Montreal Cognitive Assessment; *ABR* Auditory Brainstem Response; *AMLR* Auditory Middle Latency Response

Screening and Baseline measurements as well as treatment start can be performed on the same day. In this case, all measurements are only performed once. The baseline should be maximum 4 weeks before the treatment start; otherwise, baseline measures should be repeated (without ESIT-SQ, TSCHQ, BFI-2, ATAQ, electrophysiological measurements). ^a^Declaration of consent (ICF) can be digital for the pre-screening. ^b^Only for participants who were allocated to a single or combinational treatment with HA. ^c^Blood samples can be taken at any time point before treatment start

#### Pre-screening

Due to the investigation of a rather large and specific sample of the tinnitus population, a pre-screening will precede the actual screening process. This pre-screening is conducted as an additional layer of the eligibility assessment in the form of an online-based survey and designed to ensure a more efficient eligibility check (for an overview of study eligibility criteria see Table 1). Access to the pre-screening survey will be provided via a URL link to interested candidates. Before start of the pre-screening, candidates will be provided with information about study aim, procedure, and participation requirements. Hereinafter, participants have to confirm that they read and understood the provided information plus that they are willing to participate in the pre-screening process and answer a series of tinnitus- and health-associated questions. After completion of the pre-screening, patients will get a six-digit code, have to contact the respective study center, and leave their patient-code and phone number. Within 7 days, patients will get their results about eligibility and, if applicable, information about the subsequent screening assessment. Eligible candidates who accept the screening assessment invitation will be sent a Patient Information Leaflet and Informed Consent Form (ICF) by regular mail, to assure they have sufficient time to read the provided information and formulate questions ahead of the screening visit. Candidates who are not eligible to participate in the study will receive suggestions for treatment options outside the UNITI study.

#### Screening

Prior to start of the screening, participants will be informed about purpose, procedure, and potential risks or benefits of the trial by a member of the local study team and have to give written informed consent. Consent for the participation in the genetic tests is independent from the consent to participation in the clinical trial. Informed consent forms plus information sheets for the RCT and the blood analysis can be found in the supplemental material. Screening will be conducted at the respective clinical sites. During screening, inclusion and exclusion criteria (cf. Table 1) are verified in a clinical face-to-face setting. Several exclusion criteria are checked once again to verify that there were no changes as compared to pre-screening. In the course of the screening process, participants undergo a set of otological, audiometric, and tinnitometric examinations and have to complete several tinnitus- and health-related questionnaires. In addition, their cognitive abilities will be assessed with the MoCA test [52].

#### Baseline

After a successful screening visit and a signed ICF, several baseline measures consisting of tinnitus- and health-related questionnaires and electrophysiological measurements are conducted. Following baseline measurements, participants are randomly allocated to a treatment group (please see section “Stratification and randomization” below) and informed about the specific treatment they receive.

#### Stratification and randomization

**Fig. 2:**
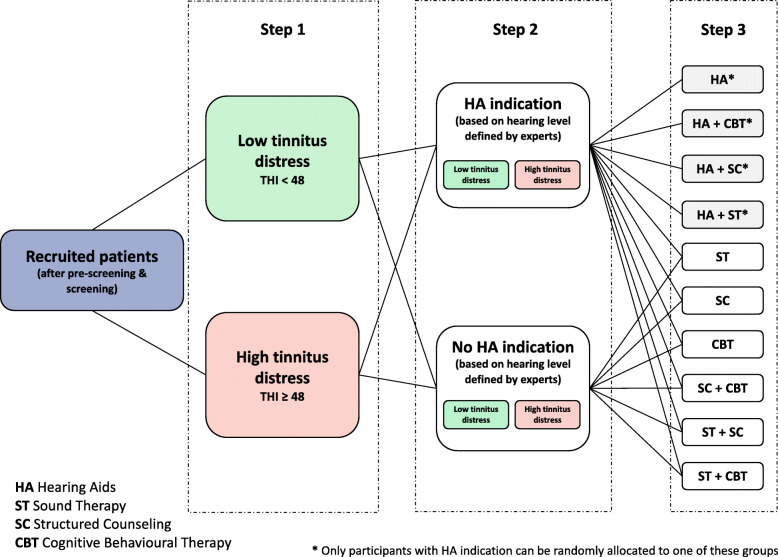
Randomization. Eligible participants will be stratified according to their level of tinnitus distress and their level of hearing loss. In a first step, stratification will be conducted based on the THI score into a low and high tinnitus distress group. In the following step 2, participants’ hearing level will be used for stratification into one group with and one group without a hearing aid indication. Patients, who are already wearing a hearing aid for 3 months before screening are automatically allocated to the no hearing aid indication group. In a final step, participants will be randomly allocated to a treatment arm comprised of single or combinatory interventions

Participants will be randomly assigned to one treatment arm comprised of a single or combinational type of intervention as exemplified in Fig. 2. Stratification will be conducted on the basis of two criteria determined during screening. As a first step, participants will be stratified according to the degree of tinnitus severity (Fig. 2, step 1). Based on their THI score [41], participants will be allocated to a low (score < 48) or a high (score ≥ 48) tinnitus distress group. Subsequently, the decision will be made, whether there is an indication for the application of a HA. Accordingly, the two subgroups of low and high tinnitus distress participants will be stratified into two groups as indicated in step 2 of Fig. 2: participants with an HA indication and those without an indication. If eligible participants have already worn a HA 3 months before screening, they will be automatically allocated to the stratification group of no HA indication. An equal ratio between the four strata (HA yes, THI ≥ 48; HA no, THI ≥ 48; HA yes, THI < 48; HA no, THI < 48) is intended. In a final step, participants will be randomly assigned to one intervention arm (cf. Fig. 2, step 3). The randomization will be executed per clinical site and monitored centrally (see section “Data management and study monitoring”). A specific interactive web response system (IWRS) will help investigators at the clinical sites to randomize their patients. This facilitates the management of a large number of patients from different sites located in several countries and the monitoring of the multicentric study with a complex design. The distribution across the four strata will be centrally monitored during the randomization process. If a recruited and eligible participant quits the RCT participation before randomization, this participant will be considered as a screening failure. In case an eligible participant is already randomized to a treatment group and quits study participation, this patient will be considered as a drop-out. The aim is to have 100 patients randomly allocated to a treatment group per clinical center.

#### Interim assessment

After 6 weeks of treatment, participants are required to make one interim visit at the clinical site in order to complete several health- and tinnitus-related questionnaires. Comorbidities, concomitant medication, and treatments as well as potential adverse events are recorded. In case of adverse events directly related to the treatment, the responsible principle investigator has to make a decision about discontinuation of the intervention. This can happen at any time during the treatment phase, since participants will be instructed to get in contact with the staff of the respective clinical site in case of adverse events. This interim visit can also be conducted online via the UNITI database in order to reduce clinical visits. Patients will get a URL link to fill out the corresponding questionnaires. Comorbidities, concomitant medication, and treatments as well as potential adverse events will be assessed telephone call.

#### Final assessment/ end of treatment

After 12 weeks, the treatment phase ends. Health- and tinnitus-related questionnaires are filled out, and several audiometric and tinnitometric measurements are executed.

#### Follow-up assessments

The first follow-up visit will take place 36 weeks after the baseline visit and consists of the same measures as taken at the final assessment/end of treatment. Though, the implementation of all otological, audiological, or electrophysiological measures are not mandatory and can be decided by the clinical sites individually. An additional facultative follow-up takes place 48 weeks after the baseline visit. Same measurements are conducted as during the first follow-up.

### Data management and study monitoring

The contract research organization Excelya (www.excelya.com) will monitor the whole trial and will perform on-site visits in order to assess the progress of the study and to ensure that it is conducted, recorded, and reported in accordance with the protocol, the timelines, Good Clinical Practice, and any other applicable regulations.

Data collected at the clinical sites will be entered in the UNITI database and treated as securely as possible and protected according to the latest international guidelines. In terms of data protection, personal data will be managed according to the EU guidelines. Participants will receive a pseudo-anonymized code, which will be used during the course of the study. Personal data will be stored at the respective clinical sites and not entered in the UNITI database. General Data Protection Regulations (GDPR) from the EU apply for all UNITI mobile applications. Full details of data protection and data security can be found in the accompanying UNITI data management plan (see supplementary material). Any disclosure of study data for scientific purposes will only be made in anonymous form.

### Adverse events

Adverse events (AE) and serious adverse events (SAE) are defined according to Good Clinical Practice §3 (6, 8). Every AE during the study will be documented with respect to start/ finish date, intensity, relation to intervention, consequences for intervention, and what actions are taken (e.g., hospitalization). Any SAE occurring during the treatment phase will be recorded and reported to the local ethics committee and the coordinator of the trial within 24 h. If a patient reports an SAE, the intervention will be immediately stopped. The study coordinator together with the local principal investigator of the respective center will decide if the participant should cease the specific treatment and which further measures are taken.

#### Risks associated with participation

There is a minor risk that the interventions used could lead to an increase of subjective tinnitus symptoms like loudness or unpleasantness. From clinical experiences, temporal increases of symptoms occur in 10% of cases. In case of tinnitus deterioration or any other adverse event, participants can quit the intervention without giving reason at any time. If desired they can receive support by trained medical or psychological experts at the specific clinical site.

Since participants have to visit clinical sites several times (e.g., study visits or CBT4T groups), there might be a slightly increased risk for a COVID-19 infection. However, safety precautions at the clinical sites following all country-specific regulations and the fact that the majority of the interventions can be performed at patient’s home (HA and mobile applications) keep the risk very low; nevertheless, it cannot be excluded. Conduction of the study will be done according to the regulations of the present local hygiene plans at the respective clinical sites. As all offered treatments are well established and as their safety profile is well known, the potential benefits of participating in this study outweigh the potential risks like a short-term increase of subjective tinnitus symptoms.

### Statistical analysis

The statistical analysis plan for the study data will be finalized before all data is collected as well as before the closure of the UNITI database and will be published separately. Hence, the following statistical analysis section provides only a brief excerpt.

Statistical analysis will be performed by a dedicated data analysis team blinded to the type of intervention. This will be accomplished by pseudo-anonymizing the treatment codes, which will only be available to the center-specific investigator responsible for the randomization. The final sample size was estimated through power calculation as already outlined above, to address the main contrast of interest, that is, whether there is a statistically significant difference in the primary outcome when comparing single and combinatory intervention. Several layers of analysis will be applied, on top of descriptive statistics. As primary outcome, tinnitus improvement (change in THI score) will be compared between the single treatment and the combinational treatment arms. Each one of the outcome measures will be compared between groups, in terms of difference between baseline and end of treatment respectively final follow-up visit. On top of this, all different groups will be compared among each other. Aim of this comparison is to identify differences between each one of the groups separately. Outcome measures will be handled as previously described. In addition, patients who underwent one specific treatment, either as single or part of combinational therapy will be grouped together, formulating four groups, representing the four different interventions (ST, HAs, CBT4T, and SC) and analyzed accordingly. This analysis will estimate the effectiveness of each one of the interventions, regardless if they were provided alone or in combination with any other treatment. Furthermore, a comparison of the subgroup of patients who received an intervention alone versus the subgroup representing those who underwent the same therapy in combination with any other kind of therapy will be conducted. For example, patients of the single treatment arm, who only received HA as intervention, will be compared with patients from the combinational arm who received HA and any other treatment and so forth. Tinnitus improvement will be further compared between ear-mediated (HA, ST) and brain-mediated (CBT4T, SC) types of interventions.

Appropriate statistical methods will be applied (*t*-test, ANOVA, etc.) under the supervision of biostatisticians. Descriptive statistics will be used for the primary and secondary outcomes, as well as included covariates. Categorical variables will be compared with chi-square tests, whereas repeated measures (pre and post treatment) will be compared with paired *t*-tests or repeated measure ANOVAs, in the case of more than two groups, after testing for distributional assumptions. Non-parametric tests will be used if the assumptions of parametric tests, such as equal variance between groups and Gaussian distributions, are not met. These tests will be used to evaluate the primary outcome across timepoints (e.g., baseline, interim visit, final visit, follow-up visit). Regression strategies with random-effect models will also be applied in order to evaluate which of the factors were determinative for each one of the outcome measures. To this end, socio-demographics, as well as questionnaires quantifying depression and tinnitus-related distress collected at baseline, will be used as covariates. Several hierarchical models will be created using the *lme4* package in R. The main goal will be to identify which baseline predictors are associated with the THI score by the end of treatment. The first set of models will compare treatments with and without hearing aids (CBT vs. CBT + HA, ST vs. ST + HA, SC vs. SC + HAs). The second set of models will assess the effect of baseline predictors on the THI by the end of the treatment in single and combinatorial treatments (SC + CBT, ST + SC, ST + CBT). Next, individual treatments will be compared against each other (CBT vs. HA vs. ST vs. SC). Finally, treatments will be divided into ear-mediated (e.g., HA and ST) and brain-mediated (e.g., CBT and SC). All models will have each center encoded as a random factor. Models will be adjusted with the covariates as described above. These analyses will aim to find the important parameters for the course in each one of the domains encountered by each one of the outcome measures. Significance level will be set to 5% for all analyses. Another objective of this RCT is the validation of a specific Decision Support System (DSS) per patient. To this end, all cases will be grouped post hoc based on whether the treatment they received based on randomization happened to be identical or not to that suggested by the DSS according to their profile. The two groups will be named DSS aligned (+) and DSS aligned (−). Whether a patient enters one of the two groups will be a matter of coincidence and not subject to randomization or actual timely DSS suggestion, since this system will not be ready for the RCT start. Evaluation of the DSS will be twofold. Initially, the outcome between DSS aligned (+) and DSS aligned (−) groups will be compared with use of the outcome measures used in the RCT. Secondly, the proportion of patients with clinically significant improvement will be compared between the two groups, indicating the positive and negative prognostic values of the platform respectively.

### Ethics/ ethical aspects

The study procedure has been submitted to and approved by the local ethics committees responsible for research activities at all investigator clinical sites. Positive evaluated ethic votes for the clinical sites in Granada, Athens, Berlin, Regensburg, and Leuven (combined ethics approval for clinical sites in Germany) can be found in the supplemental material. Prior to start of the study eligible patients have to give written informed consent. Each patient will get detailed and comprehensive information about the objectives, procedures, and interventions of the RCT plus potential side effects associated with a participation as well as their right to cancel a participation at any time during the study without any reason or detriment. Participants can request that their study data are removed from the database and excluded from future analysis. All documentation that is needed for possible regulatory audits, e.g., the signed ICF, cannot be deleted, even if this is requested by the patient. Any changes required to the protocol are discussed and decided jointly in a dedicated meeting of the responsible PIs. All amendments will be prepared by the RCT coordinator team in Regensburg and submitted to all local ethics committees.

## Discussion

The present study protocol describes an international multi-center RCT for chronic tinnitus using four different types of interventions (HA, ST, SC, CBT4T), performed either as a single treatment or a combination of two treatments over the course of 12 weeks.

For many people affected, tinnitus constitutes a massive burden with many potential psychological and physiological comorbidities [1, 69, 70], ultimately resulting in lower quality of life [71, 72]. The used interventions in the UNITI-RCT might have the potential for brief or even prolonged tinnitus suppression as well as to help people to better cope respectively live with their tinnitus.

With its primary objective to evaluate not only single treatments but also the combination among them, the UNITI-RCT will be the first proper systematic RCT in tinnitus to scrutinize single and combinatory interventions in more depth. Given the fact that numerous single-target studies have failed to establish an effective therapy, the combination of different types of interventions allows a targeting of different involved organ levels at the same time (AS, CNS) and might manifest in better treatment responses.

Furthermore, the UNITI-RCT attempts to overcome essential limitations of previous studies [20, 21] through a large sample size, harmonized patient selection and screening processes across the five participating clinical centers, standardized assessments methods, and interventions across the clinical sites, and large-scale data analysis strategies. Previous reviews of tinnitus treatments provide evidence that only three past RCTs exceed 250 participants [23]; to the best of our knowledge, the currently largest RCT included 492 patients [18]. With the aim to investigate 500 tinnitus patients, the UNITI-RCT has the attempt to be the largest RCT in tinnitus yet. Importantly, with the UNITI-RCT, we also include the combination of treatments.

Due to tinnitus heterogeneity [24] as well the lack of clarity about pathophysiological processes [73, 74], up-to-now there is no cure for tinnitus [3, 75]. In the majority of studies, only a subgroup of tinnitus patients demonstrates improvement [23]. It has been proposed that precision medicine approaches should be tackled by future tinnitus research [21, 24]. The UNITI-RCT aims at making a significant contribution to the personalization of tinnitus treatments by means of the validation of a specific DSS. The system will be developed during the UNITI project [38], in order to support clinicians in choosing the optimal treatment based on an individual patient’s profile [39]. Such a system and its underlying computational model has not only the potential to significantly contribute to our understanding of the tinnitus pathology, but also improve clinical practice and the way we are trying to treat tinnitus.

It has already been suggested that mobile applications offer a promising way for tinnitus assessment and treatment [76–78]. Hence, several specific mobile applications will be developed for the UNITI-RCT creating the opportunity to significantly reduce the number of tinnitus-related clinical visits and as such the necessary health care resources. Moreover, these mobile applications do not only provide an ecological way to evaluate patients’ tinnitus at many different timepoints with the possibility to track daily tinnitus fluctuations and detailed treatment-related consequences, but also provide maximum flexibility in space and time from a patients’ view—potentially increasing compliance.

Taken together, the UNIT-RCT with its multidisciplinary multi-center approach, standardized state-of-the-art tinnitus interventions and assessments, novel treatment combinations, and mobile applications as well as its major contribution to personalize tinnitus treatments might not only represent a new methodological benchmark in tinnitus trials but also provide an essential step towards a cure for tinnitus.

## Status of the trial

Issue date: 04. November 2021. Protocol version no. 3. The first stage of patient recruitment started in March 2021 with the online pre-screening. In April 2021, screening and baseline measurements started at the clinical sites in Berlin and Regensburg. In June 2021, these measurements started at the clinical site in Granada and Athens as well. The trial including all mandatory assessments at all involved clinical sites is due to be finished by the end 2022.

## Supplementary Information

**Additional file 1.** Ethical approvals from Germany, Spain, Greece and Belgium. Informed consent form – RCT. Information sheet – RCT. Informed consent form – blood sampling. Information sheet – blood sampling. UNITI data management plan. WHO trial registration dataset.

## Data Availability

Not applicable

## Abbreviations

ABR: Auditory brain stem response
AE: Adverse event
AMLR: Auditory middle latency responses
ANOVA: Analysis of variance
AS: Auditory system
ATAQ: Attitudes Towards Amplification Questionnaire
BFI-2: Big Five Inventory-2
CBT4T: Cognitive behavioral therapy for tinnitus
CGI-I: Clinical Global Impression Scale – Improvement
CNS: Central nervous system
DNA: Deoxyribonucleic acid
DSS: Decision support system
ESIT-SQ: European School of Interdisciplinary Tinnitus Research Screening Questionnaire
EU: European Union
FTQ: Fear of Tinnitus Questionnaire
GDPR: General Data Protection Regulations
GUF: Questionnaire on Hypersensitivity to Sound
HA: Hearing aid
ICF: Informed consent form
Mini-TQ: Mini Tinnitus Questionnaire
MoCA: Montreal Cognitive Assessment
PEA: Proximity extension assay
PHQ-D: Patient Health Questionnaire for Depression
RCT: Randomized clinical trial
SAE: Serious adverse event
SC: Structured counseling
SNV: Single-nucleotide variants
SOISES: Social Isolation Electronic Survey
ST: Sound therapy
TFI: Tinnitus Functional Index
THI: Tinnitus Handicap Inventory
TSCHQ: Tinnitus Sample Case History Questionnaire
UNITI: Unification of Treatments and Interventions for Tinnitus Patients
WhoQol-BREF: World Health Organization Quality of Life - abbreviated
