# Supplementary material for: Unification of Treatments and Interventions for Tinnitus Patients (UNITI): a study protocol for a multi-center randomized clinical trial
Source: Trials. 2021 Dec 4;22:875. doi: 10.1186/s13063-021-05835-z (PMC8642746; doi:10.1186/s13063-021-05835-z)
Supplement: Supplementary file 1 — Additional file 1. Ethical approvals from Germany, Spain, Greece and Belgium. Informed consent form – RCT. Information sheet – RCT. Informed consent form – blood sampling. Information sheet – blood sampling. UNITI data management plan. WHO trial registration dataset. [file 13063_2021_5835_MOESM1_ESM.zip › Amendmend1_ethical_approval_Berlin_RegensburgR1.pdf]

Universität Regensburg

Ethikkommission
bei der Universität Regensburg

Ethikkommission · Universität Regensburg · 93040 Regensburg

Prof. Edward K. Geissler, PhD, Vorsitzender

Dr. iur. Frederike Seitz, M.A., Geschäftsführerin

Geschäftsstelle:
Telefon +49 941 943-5370
Telefax +49 941 943-5369
Postanschrift:
Universität Regensburg
ETHIKKOMMISSION
D-93040 Regensburg

ethikkommission@ur.de
http://ethikkommission.uni-regensburg.de

Bezirksklinikum Regensburg, Klinik und
Poliklinik für Psychiatrie und Psychotherapie
Stefan Schoisswohl, BSc MSc
An der Steinernen Bank
93080 Pentling
Deutschland

15.03.2021

Unser Zeichen: 20-1936_1-101

Beratung nach § 15 Abs. 1 Berufsordnung für die Ärzte Bayerns – Nachträgliche Änderung vom *11.03.2021*

für das

**Forschungsvorhaben**	**UNification of treatments and Interventions for TInnitus patients – Randomized Clinical Trial (UNITI-RCT)**
Antragssteller	Stefan Schoisswohl, BSc MSc

Die Ethikkommission nimmt die nachträglichen Änderungen am o.g. Forschungsvorhaben zur Kenntnis. Eine erneute inhaltliche Bewertung ist nach geltendem Recht nicht vorgesehen.

Diese Entscheidung erging durch den Vorsitzenden der Ethikkommission im Benehmen mit der Geschäftsstelle im beschleunigten Verfahren.

Es wird auf folgendes grundsätzlich hingewiesen:

Die ärztliche und juristische Verantwortung verbleibt beim Forscher und seinen Mitarbeitern.

Die Auflagen der Deklaration von Helsinki des Weltärztebundes in ihrer aktuellen Fassung hinsichtlich ethischen und rechtlichen Aspekten biomedizinischer Forschung am Menschen sind strikt zu beachten.

Die Ethikkommission erwartet bei Interventionsstudien, dass ihr alle schwerwiegenden oder unerwarteten unerwünschten Ereignisse (u.a. Todesfälle), die während der Studie auftreten und die Sicherheit der Studienteilnehmer oder die Durchführung der Studie beeinträchtigen können, unverzüglich schriftlich mitgeteilt werden. Dieses sollte in Verbindung mit einer Stellungnahme des Antragsstellers geschehen, ob aus seiner Sicht die Nutzen-Risiko-Relation des Vorhabens verändert ist.

Die Ethikkommission bittet darum, dass ihr der Abbruch oder Abschluss einer Studie mitgeteilt werden.

Dieses Schreiben ist mit den Studienunterlagen jederzeit sorgfältig aufzubewahren. Duplikate oder Abschriften dieses Schreibens können im Nachhinein nicht erstellt werden.

Auf die Rechtspflichten zum Umgang mit dienstlichem Schriftgut bzw. Urkunden wird verwiesen.

Die Ethikkommission bestätigt die Bearbeitung gemäß der GCP/ICH-Richtlinien.

Die Ethikkommission empfiehlt im Einklang mit der Deklaration von Helsinki nachdrücklich die Registrierung der Studie vor Studienbeginn in einem öffentlich zugänglichen Register, das die von der WHO geforderten Voraussetzungen erfüllt.

Falls kein gesetzlicher Kostenbefreiungstatbestand greift, wird ein gesonderter Kostenbescheid für die Gebühren und Auslagen der Ethikkommission ergehen.

Die Übermittlung personenbezogener Daten einschließlich DNA-tragender Biomaterialien in datenschutzrechtlich unsichere Drittstaaten, wie etwa die USA, bedarf einer gesonderten datenschutzrechtlichen Beurteilung und Risikoaufklärung.

Datenschutzrecht wird durch die Ethikkommission grundsätzlich nur kursorisch geprüft. Dieses Votum ersetzt mithin nicht die Konsultation des zuständigen Datenschutzbeauftragten.

Mit dem Urteil des Europäischen Gerichtshofs vom 16. Juli 2020 [Aktenzeichen C3-11/18] stellen die Regelungen des EU-US-Privacy Shield insbesondere vor dem Hintergrund des Clarifying Lawful Overseas Use of Data Act (CLOUD Act) bzw. des Foreign Surveillance Act (FISA) keinen tauglichen Rechtsrahmen mehr dar. Es sollte seitens der Verantwortlichen im Einzelfall geprüft werden, inwieweit personenbezogene/personenbeziehbare Daten (also auch i.S.d. Art. 4 Abs. 5 DSGVO pseudonymisierte Datensätze) rechtssicher entweder auf Basis geeigneter Garantien (etwa verbindlicher Unternehmensregeln, Standardvertragsklauseln oder auf Basis einer ausdrücklichen Einwilligung nach erfolgter Risiko-Aufklärung nach Art. 49 Abs. 1 lit. a) DSGVO) übermittelt werden können. Es bleiben v.a. hinsichtlich der Standardvertragsklauseln die Auswirkungen des Urteils und die voraussichtlich folgenden regulatorischen Leitlinien seitens der zuständigen Behörden aufmerksam zu verfolgen. Es ist daher den Sponsoren dringend zu raten, sich mit dem zuständigen Landesbeauftragten für den Datenschutz abzustimmen.

Mit freundlichen kollegialen Grüßen

Prof. Edward K. Geissler, PhD
Vorsitzender

Anlage

Dokumente betreffend diesen Vorfall:

Anlageelemente	Datei-Name	Datum
Keine	amendment_UNITI_vers2.pdf	12.03.2021
Keine	clinical_investigation_plan_UNITI_vers2_amend1.pdf	12.03.2021
Keine	UNITI_blood_Einverständniserklärung_final.pdf	12.03.2021
Keine	UNITI_blood_Informationsblatt_final.pdf	12.03.2021
Keine	UNITI_Einverständniserklärung_final.pdf	12.03.2021
Keine	UNITI_eligibility_checklist_vers2_amend1.pdf	12.03.2021
Keine	UNITI_Informationsblatt_ver6_amend1.pdf	12.03.2021
