# Supplementary material for: Unification of Treatments and Interventions for Tinnitus Patients (UNITI): a study protocol for a multi-center randomized clinical trial
Source: Trials. 2021 Dec 4;22:875. doi: 10.1186/s13063-021-05835-z (PMC8642746; doi:10.1186/s13063-021-05835-z)
Supplement: Supplementary file 1 — Additional file 1. Ethical approvals from Germany, Spain, Greece and Belgium. Informed consent form – RCT. Information sheet – RCT. Informed consent form – blood sampling. Information sheet – blood sampling. UNITI data management plan. WHO trial registration dataset. [file 13063_2021_5835_MOESM1_ESM.zip › UNITI_certificate_of_consent_bloodsamplingR1.pdf]

PART 2: CERTIFICATE OF CONSENT – Genetic Analysis

Herewith I

Last Name/ First Name: ……………………………………………………………………………………………….

Date of birth: ……….

Address: …….

Phone number/ email: ………………………………………………………………………………………….

agree to give a blood sample for genetic analysis in the course of the study “**Unification of treatments and Interventions for Tinnitus Patients**” at the <<insert name of clinical site>>.

I hereby confirm that I have been fully informed or educated about the genetic analyses carried out in the UNITI study.

I confirm that I have read the patient information sheet on genetic research and understand its contents. I had enough time to make a decision and had the opportunity to ask additional questions about participation in the research project.

I understand that my participation and sample donation is voluntary and that I may withdraw my consent at any time without affecting my future care.

I agree that the data collected in the course of the genetic research, including the information collected in the UNITI study (e.g. about my tinnitus, my state of health and response to treatment), may be used as described in the genetic research information sheet as part of this consent form. This does not waive any rights I may have under current legislation.

I consent to this data being processed and analyzed in the laboratories belonging to the UNITI Study Consortium (Stockholm, Sweden, Karolinska Institutet and Granada, Spain, Centre for Genomics and Oncological Research GENYO), as described in the section "How will the genetic data and my personal data be used and protected?

I understand that in order to verify the proper conduct of genetic research, the medical study records may be inspected by the relevant regulatory authorities. I will grant access to the records to those responsible.

I agree that the results of this genetic study may be shared by the study team/principal investigator (PI) with other researchers in summary form (study code) for future health research purposes.

I understand that after signing, I will be given a copy of this consent form and a copy of the patient information sheet.

I agree that the genetic sample I donate and the data collected in the UNITI study may be used as indicated in this document.

……………………………………………… ……………………………………………………………………………

Place/ Date **Participant´s signature**

The study consent conversation was carried out by: ………………………………………………………………

Herewith I declare, that methods, aim and procedure as well as potential benefits and risks were fully and comprehensively explained to the above-mentioned participant on ………………………. (dd-mm-yyyy), in both verbally and in written form. I also confirm to have handed over a copy of the study information sheet as well as a copy of this signed consent form to the participant.

……………………………………………… ……………………………………………………………………………

Place/Date **Study team member´s signature**

Contact information:

<<insert contact information>>
