# Supplementary material for: Unification of Treatments and Interventions for Tinnitus Patients (UNITI): a study protocol for a multi-center randomized clinical trial
Source: Trials. 2021 Dec 4;22:875. doi: 10.1186/s13063-021-05835-z (PMC8642746; doi:10.1186/s13063-021-05835-z)
Supplement: Supplementary file 1 — Additional file 1. Ethical approvals from Germany, Spain, Greece and Belgium. Informed consent form – RCT. Information sheet – RCT. Informed consent form – blood sampling. Information sheet – blood sampling. UNITI data management plan. WHO trial registration dataset. [file 13063_2021_5835_MOESM1_ESM.zip › UNITI_certificate_of_consent_RCTR1.pdf]

PART 2: CERTIFICATE OF CONSENT – UNITI-RCT

Herewith I

Last Name/ First Name: ………………………………………………………………………………………………

Date of birth: ………

Address: ……

Phone number/ email: ………………………………………………………………………………………………

agree to participate in the study "**Unification of treatments and Interventions for Tinnitus patients**" at the <<insert name of clinical site>>.

In doing so, I will be randomly assigned to an intervention for tinnitus consisting of single or combinational types of treatment. After one-three screening/baseline visits, I will receive a 12-week long treatment for tinnitus. I agree to complete several tinnitus- and health-related questionnaires as well as clinical measures at up to five different visits with a duration of about 2 hours each (in case screening and baseline are performed on the same day – up to 4 hours).

I hereby confirm that I have been fully informed about the implementation of the UNITI study.

I confirm that I have read and understood the Patient Information Sheet on the conduct of the UNITI study. I had enough time to make a decision and had the opportunity to ask additional questions about participating in the study.

I understand that my participation is voluntary and free of charge, and that I can withdraw my consent at any time without giving any reason, without this having any effect on my future care.

I confirm that I am able and willing to fully complete my participation in this study.

I understand that I must inform the study doctor or the responsible staff before I take a new drug or start a new treatment.

I grant authorized representatives or national/international health authorities (e.g. European Medicines Agency/EMA, US Food and Drug Administration/FDA or national health authorities) free access to my medical files for quality assurance of the clinical trial, provided that my data remain confidential and secret.

I understand that neither I nor my public or private insurance company will incur any costs from the treatment, procedures and tests of the study.

I agree that my study data may be used and shared by the study team/principal investigator (PI) and other researchers for additional research in an anonymous form, without the possibility to draw conclusions about personal information.

I have been informed about the protection of my personal data.

- ▢ I do not want the study doctor to tell my personal doctor that I am taking part in this study.

I understand that after signing, I will be given a copy of this consent form and a copy of the patient information sheet.
I have decided to participate in this study.

……………………………………………… ……………………………………………………………………

Place/Date **Participant´s signature**

The study consent conversation was carried out by: ……………………………………………………………

Herewith I declare, that methods, aim and procedure as well as potential benefits and risks were fully and comprehensively explained to the above-mentioned participant at the ……………………… (dd-mm-yyyy) in both verbally and written form. I also confirm to handed over a copy of the study information sheet as well as a copy of this signed consent form to the participant.

……………………………………………… ……………………………………………………………………

Place/Date **Study team member´s signature**

Contact informations:

<<insert contact informations>>
