# Supplementary material for: Unification of Treatments and Interventions for Tinnitus Patients (UNITI): a study protocol for a multi-center randomized clinical trial
Source: Trials. 2021 Dec 4;22:875. doi: 10.1186/s13063-021-05835-z (PMC8642746; doi:10.1186/s13063-021-05835-z)
Supplement: Supplementary file 1 — Additional file 1. Ethical approvals from Germany, Spain, Greece and Belgium. Informed consent form – RCT. Information sheet – RCT. Informed consent form – blood sampling. Information sheet – blood sampling. UNITI data management plan. WHO trial registration dataset. [file 13063_2021_5835_MOESM1_ESM.zip › UNITI_data_management_planR1.pdf]

Unification of treatments and interventions for tinnitus patients

Proposal No.: 848261

Deliverable D2.1:

Data Management Plan

Deliverable No. D6 – WP2

Authors	Georgios Spanoudakis (SPX), Susanne Staudinger (UHREG), Winfried Schlee (UHREG)
Responsible of Deliverable	Winfried Schlee (UHREG)
Target Dissemination Level	Public
Status of the Document	Version 3, February 21, 2020

Table of contents

1 Introduction ... 3

2 Data handling during the project ... 4

2.1 Unified database for clinical data ... 4

2.2 Unified database for smartphone data ... 6

2.3 Matching data from the EU Tinnitus Database with the app data ... 7

3 Data handling after the project and openly accessible data ... 8

4 Conclusions ... 10

1 Introduction

Tinnitus is a frequent clinical condition that can have a negative impact on the affected individual in multiple domains of everyday life. Tinnitus remains a scientific and clinical enigma of immensely high prevalence and socio-economic burden. It can occur in all ages, in various frequencies, intensity and duration scales, whereas annoyance caused to the patients varies from totally absent to tinnitus-related suicidal tendency.

Tinnitus is a heterogeneous condition and complex in many ways. Its pathophysiology has not been resolved, since deterioration of cochlear elements, synapses, certain Central Nervous System (CNS) anatomical sites, and abnormal communication patterns, inhibition of neuro-transmitters, behavioural diversities are only some of the models suggested, but none enjoys common acceptance or integrated results in the clinical level. In addition, a large variety of patient characteristics are poorly understood, because integrated systems approaches are still missing to correlate patients' characteristics to predict responses to combinatorial therapies.

No single self-report instrument captures all this diversity, which poses challenges for characterising tinnitus and making informed treatment decisions. It is therefore necessary to collect clinical information of the patients using several measuring instruments and converge the information into a coherent picture of the individual patient. Only a large collection of data, together with the results from clinical interventions, can inform computational models in order to retrieve knowledge about which treatment works best for which type of patient. This is the aim of the UNITI project.

This deliverable constitutes the data management plan of the project and describe how the data that will be collected for the purposes of UNITI will be handled during the project and after the end of it following the international standards and concepts of the FAIR principles. It should be noted that despite having taken all necessary steps to ensure that the current data management plan covers comprehensively all data management issues which might arise in UNITI, there is a possibility to encounter a need to revise it. If such a need arises during of the project, the data management plan will be amended and communicated to all stakeholders, who should be notified about the amendments.

The reminder of this deliverable is structured as follows:

- Section 2 summarises the handling of data during the project
- Section 3 summarises the handling of data after the end of the project
- Section 4 provides concluding remarks.

2 Data handling during the project

2.1 Unified database for clinical data

Introduction: The EU Tinnitus Database will be used for the storage of clinical data whithin the UNITI project. Historically, the EU Tinnitus Database is based on the ESIT Database which is used in the H2020 project ESIT and hosted by the tinnitus group from the University of Regensburg.

Types of data: The following types of data are stored in the EU Tinnitus Database: Medical and tinnitus-related history; audiological examinations; questionnaire data. It also allows uploading and storing data files for individual patient.

Sources of data: The EU Tinnitus database is fed with data collected by the individual clinical partners.

Ownership of data: The owner of the data is always the Centre where the data was collected. This centre is represented by the principal investigator who is responsible for it.

Pseudonymisation of data: The EU Tinnitus database does not store personal information like names, addresses, phone numbers, e-mail addresses, or IP addresses that can be used to identify a certain participant directly. A system of two-level pseudo identifiers will be used to anonymise the data. The first pseudo identifier (PSID1) will be generated in the system(s) by the system that is the source of the data (i.e., it generates them or it is used to record them in the first place). Data communicated to the unified UNITI database will have PSID1 but before stored in it, PSID1 will be replaced by a second pseudo identifier (PSID2) which will be generated by the UNITI database. Subsequently, data will be recorded with PSID2. PSID1 will PSID1 and PSID2 will be used to link and combine data from different sources. In cases where the data are inserted directly into the UNITI database only PSID2 will be generated from the real identifier.

To enable responses to requests made under GDPR, the association between the real identifier of a patient and PSID1 (PSID2) will be maintained in encrypted form at the source system that provided the data for the particular patient. The association between PSID2 and PSID1 (when necessary) will also be maintained separately and in encrypted form. These associations will be accessible only to authorised and authenticated software components of the UNITI platform and users with appropriate authorisation rights, who may need to access the real identity of a patient.

Furthermore, when the data are exported for further analysis, PSID2 is removed and replaced by a random unique identifier. All types of data analysis can only be executed upon the anonymised data set.

Personal information of individual patients is maintained only within the system of the local centre, which has responsibility for the patient and, therefore, needs to identify the patient. If a patient can be identified individually, e.g. by the value of the external identifier attribute, and requests for their data to be anonymised or deleted according to the rules set by the General Data Protection Regulation (GDPR), the database administrative staff of the local systems of the centres anonymise or delete the participants data permanently, as requested.

Data quality: The first step taken to ensure high-quality data within this framework was to implement the user interface using standardised input fields that can be used to assist and restrict user input where reasonable. One example is the use of standardised inputs for integral values that will report ill-formed input to the user automatically. Whenever a participant decides to save their current progress, the data are validated on the server side and the results will be reported to the user in different ways. For every validation error, the

corresponding question is highlighted and the part that includes the error is indicated. Furthermore, the overall state of data entry is indicated with localised status messages and textual instructions.

Languages: The database offers support for different languages. These include Dutch, German, Greek, Italian, Polish, Spanish, and Swedish. The available languages cover all languages of the UNITI clinical centres, plus additional languages. The language settings of the database can be changed by the different users.

Web access: The database can be reached via www.tinnitus-database.eu. Only contributors and members of the EU Tinnitus Database have access to it.

Database manager: The database will be managed by Jorge Simoes from UHREG who is overseeing the database, ensuring the maintenance, the data handling plan, data quality and further developments, if needed.

Role based access control. Different levels of access to the database are provided subject to the roles of individual users. Users are members of staff of the different clinical centres. In general, users are researchers and they can only see the data of their own centre. At the moment, the roles supported (Clinical) Centre Editors, Experts and Administrators. Role based access control is enforced by a role management system, allowing Centre Editors to view and edit individual patient datasets of their respective centre. In addition to the rights of Centre Editors, Centre Experts can export data of all the patients of their centre for data analysis. Centre Administrators have all rights of the Centre Editors and Centre Experts but they can also add new users to the database or remove users from it. There is also a database administrator user (Superadmin) based at the University of Regensburg, who has access to the data from all centres. Our expectation is that the above role types will remain in UNITI and may be expanded by other additional roles (e.g., Centre admin users with enhanced rights.

Local data analysis: Local data analysis means a data analysis that is based on the data set that has been collected at the Centre that is performing the data analysis. The person who is responsible for the local data analysis is identical to the owner of the data. Individual centres (i.e., clinical partners in UNITI) can perform data analysis on their own data. This right arises from their role.

Multi-centre analysis: The standardised data assessment and storage in the EU Tinnitus database allows efficient multi-centre data analysis of the participating partners. Multi-centre analysis means a data analysis that is based on data that have been collected by two or more partner centres. If one or more partner centres plan to perform a multi-centre data analysis, they need to contact the owners of the datasets that will be included in the envisaged analysis. An agreement on the aim of multi-centre data analysis and the authorships for the paper that might result of the analysis need to be settled and signed by all data owners. After sending this data analysis agreement to the database administrator, the administrator provides access to the respective data set as determined by the agreement. Multi-centre analysis can only be applied to anonymised datasets.

Data Handling Plan. A data handling plan has been developed as a manual for understanding and analysing the data stored in the EU Tinnitus Database that will be included in the UNITI database. This plan gives and overview of how to interpret data, how questionnaire items are coded, how missing values are coded, the rules for calculating sum scores of the clinical questionnaires and all other information that are needed for unified interpretation of the data. The data handling plan is written in English and is accessible to all contributors via the EU Tinnitus Database in a dedicated download section. The data

handling plan is kept up to date by UHREG using a revision numbering system. The database adminsitrator is overseeing the updates of the data handling plan.

Data protection: Data protection is considered and relevant ethical, legal and privacy concerns will be addressed respectively. The Data Protection Officer (DPO) of the University of Regensburg (Germany) is also the Data Protection Officer of the EU Tinnitus Database. He is responsible for overseeing the data protection strategy and implementation to ensure compliance with the EU General Data Protection Regulation (GDPR, 2016/679). Furthermore to ensure that the project will be able to respond to data subject access requests (DSA) under GDPR associations between the pseudo identifiers and real identifiers of subjects will be maintained separately from the clinical data, in encrypted form and in

Data transfer: The rules for the secure data transfer within the UNITI consortium is regulated by UNITI Consortium Agreement under chapter 12.

Data export and data analysis: Within the internal section of the database, the staff can monitor and review data entry and export the data when needed. Authorised staff can configure custom selection criteria depending on analysis or study requirements. For example, the data export can be configured to exclude datasets that are not fully validated, are missing certain items, or meet other criteria for exclusion. Each data export will be automatically recorded in a log file, that contains all configuration settings by the user and a time stamp. This allows reconstruction of the data export, if needed. The data will be exported without any personal information of the participants and the pseudonymization code will be removed automatically. Therefore, all data analysis will be performed on so-called anonymized data sets. The data export will be saved in a CSV file, with horizontal or vertical data format. The CSV file is readable by all major statistical software packages. The majority of data analyses will be executed using the open source statistical software package R (www.r-project.org). However, each researcher is free to use the statistical software of choice.

Physical database: The data is stored in a relational database format using Maria DB 11 (www.mariadb.org), which runs in a Linux environment (Debian Buster, www.debian.org/releases/buster/) and a LAMP technology stack (www.whatis.techtarget.com/definition/LAMP-Linux-Apache-MySQL-PHP).

Backup: A backup of the database is performed every night for data security reasons whereby the database is backed up to a server hosted by the Strato AG (Berlin, Germany) and to a second server located at the DBIS institute. All servers are located in Germany. A Secure Sockets Layer (SSL) protocol is used for all data transfers.

2.2 Unified database for smartphone data

Introduction: The unified database for smartphone data has the purpose to store the data that is collected with the smartphone apps within UNITI.

Types of data: The following types of data are stored in the app database and the local smartphone devices:

- questionnaire data collected using Ecological Momentary Assessment (EMA) methodology;
- time stamps of app usage;
- in case of auditory stimulation: subjective ratings of the tinnitus suppression;
- in case of psychoeducation app: results of the quiz;
- in case users allow it: GPS location and sound pressure of the surrounding environment while filling out the EMA questionnaires.

Sources of data: The smartphone device, its sensors and the UNITI app running on it.

Ownership of data: The patient who uses the smartphone device and UNITI app.

Physical database: The data collected from the smartphone is stored in a relational database using Maria DB 11, which runs in a Linux environment (Debian Buster) and a LAMP technology stack. This database will be separate from the UNITI database and may be referred to as the "smartphone database". The smartphone database will only be accessible to the patients through the UNITI mobile app running on the smartphones of the patients. Access to it will also require the authentication of the particular UNITI mobile app instance as well as the authentication of the user of this app (the patient). Smart phone data will also be stored separately in the UNITI database for analysis purposes.

Pseudonymization of data: The smartphone database does not store personal information like names, addresses, phone numbers, e-mail addresses, or IP addresses that can be used to identify a certain participant directly. Data from the smartphone will be stored in the smartphone database and the UNITI database separately after pseudo-anonymisation as described in Section 2.1. Furthermore, on the smartphone database, data will be stored in encrypted form.

Data quality: Checks similar to those described for the UNITI database will also be implemented for the mobile app in order to ensure the quality of the data collected by it.

Languages: The mobile app offers support for different languages, as described for the UNITI database.

Access of data: The patients can see their own data on their smartphone device. Such data will be maintained in encrypted form on the mobile accessible only after proper authentication of the patient/user of the mobile. This authentication will be based on password or fingerprint authentication. Mobile app data will also be transferred and backed up periodically on a back-end server of the UNITI platform (separate from the UNITI unified database), to ensure availability in the event of loss of the mobile device and to ensure that the storage space on the device will always be sufficient for storing the latest data of the patient. Backed up data will be stored in encrypted form and will be accessible only to the client app running on the mobile device. Users of the UNITI unified database will not have access to such data (although they will have access to their pseudo-anonymised counterparts).

2.3 Matching data from the EU Tinnitus Database with the app data

A unique patient identifier will be used in the EU Tinnitus Database as well as for the smartphone apps. This identifier will be used to match the data from the two SQL databases.

3 Data handling after the project and openly accessible data

3.1 Publication data

We envisage that a repository for depositing publications and data related to them will be needed. Zenodo (zenodo.org) is a good candidate for such repository and it will be considered further as a candidate repository for publication data. Furthermore, the DataCite Metadata Schema (schema.datacite.org), will be adopted in this case.
Furthermore, UNITI advocates the "FAIR" principles[1] with regards to supporting open access to publication data and anonymised patient data. The FAIR principles require data to be findable (F), accessible (A), interoperable (I) and re-usable (R). These principles precede implementation choices and do not necessarily suggest any specific technology, standard, or implementation-solution. The measures that we will take to support these principles are summarised below.

Findable data

F1: (meta)data are assigned a globally unique and persistent identifier

- A persistent and unique Digital Object Identifier (DOI) is issued to every published record. Moreover, DOI versioning is supported and enables users to update the record's files after they have been made public and researchers to easily cite either specific versions of a record or to cite, via a top-level DOI, all the versions of a record.

F2: data are described with rich metadata (defined by R1 below)

- The repository's metadata schema will be compliant with DataCite's Metadata Schema minimum and recommended terms, with a few additional enrichments. As there are no specific metadata schemas that can be used with the UNITI data this more generic schema will be adopted.

F3: metadata clearly and explicitly include the identifier of the data it describes

- The DOI is a top-level and a mandatory field in the metadata of each record.

F4: (meta)data are registered or indexed in a searchable resource

- Metadata of each record will be indexed and searchable directly in the repository's search engine immediately after publishing.
- Metadata of each record is sent to DataCite servers during DOI registration and indexed there.

Accessible data

A1: (meta)data are retrievable by their identifier using a standardized communications protocol

- Metadata for individual records as well as record collections are harvestable using the OAI-PMH protocol by the record identifier and the collection name.
- Metadata is also retrievable through the public REST API.

A1.1: the protocol is open, free, and universally implementable

- See point A1. OAI-PMH and REST are open, free and universal protocols for information retrieval on the web.

A1.2: the protocol allows for an authentication and authorisation procedure, where necessary

---

[1] Wilkinson, M. D. *et al.* The FAIR Guiding Principles for scientific data management and stewardship. *Sci. Data* 3:160018 doi: 10.1038/sdata.2016.18 (2016).

- Metadata are publicly accessible and licensed under public domain. No authorization is ever necessary to retrieve it.

A2: metadata are accessible, even when the data are no longer available

- Data and metadata will be retained for the lifetime of the repository.
- Metadata are stored in high-availability database servers at ULM, which are separate to the data itself.

Interoperable data

I1: (meta)data use a formal, accessible, shared, and broadly applicable language for knowledge representation.

- Zenodo uses JSON Schema as internal representation of metadata and offers export to other popular formats such as Dublin Core or MARCXML.

I2: (meta)data use vocabularies that follow FAIR principles

- For certain terms it refers to open, external vocabularies, e.g.: license (Open Definition), funders (FundRef) and grants (OpenAIRE).

I3: (meta)data include qualified references to other (meta)data

- Each referenced external piece of metadata is qualified by a resolvable URL.

Re-usable data

R1: (meta)data are richly described with a plurality of accurate and relevant attributes

- Each record contains a minimum of DataCite's mandatory terms, with optionally additional DataCite recommended terms and Zenodo's enrichments.

R1.1: (meta)data are released with a clear and accessible data usage license

- License is one of the mandatory terms in Zenodo's metadata, and is referring to an Open Definition license, but within HOLOBALANCE restricted access will be chosen for the patient data.
- Data downloaded by the users is subject to the license specified in the metadata by the uploader.

R1.2: (meta)data are associated with detailed provenance

- All data and metadata uploaded is tracable to a registered Zenodo user.
- Metadata can optionally describe the original authors of the published work.

3.2 Patient data

The UNITI project aims towards open data. However, depending on the regulations of the local ethical committees, it might be that not all data can be made open. Our overall aim is to turn as much data as possible into open data, which will be provided only in anonymized form. To ensure the correct usage of the data that will be made open, we aim for an open access publication that will describe the data set, the interpretation of the values, the recruitment procedure and all other details that will be needed for further research on the particular open dataset.

It is foreseen, that at the end of the project, the associations between pseudo identifiers enabling the pseudonymization of patient data, described in Section 2, will be completely deleted. This will lead to full anonymisation of the acquired data set. Nevertheless, it is possible that, different regulations of the ethical committees of the different clinical partners or legal interpretations of GDPR taken at national level, might enforce an earlier or later time point of deletion.

After full anonymisation is carried out, our aim is to maintain the fully anonymised EU Tinnitus Database for at least 10 years after the lifetime of the UNITI project in order to ensure that the data are findable, accessible, interoperable and re-usable (FAIR).

Towards this direction, the UNITI consortium will also consider the possibility of offering the data set that may become open source in formats that would enable the viewing of the data through commonly used software such as video viewers and/or text editing software. For processing, it will also consider making the dataset available in formats that would enable their processing in open source data analysis software like, for instance:

- GNU Octave (www.gnu.org/software/octave/)
- Scilab (www.scilab.org)

The possibility of providing open source data through the Open Research Data (ORD) pilot initiative[2] of the European Commission will also be investigated. ORD has been created primarily with the intention to enable the provision of data needed to validate the results presented in scientific publications. It does cover however other types of data that are voluntarily offered by beneficiaries of Horizon 2020 projects. A portal making available such datasets has also been set up and maintained by the EU, i.e., the EU Open Data Portal (ODP)[3]. The use of ORD and ODP will be considered as a means of offering as open source data sets required for the validation of UNITI publications and/or wider anonymised datasets, as discussed above.

All the above actions as well as any other action that may be necessary for the purpose of providing anonymised datasets of UNITI as open source data will be taken only after approval from the UNITI management board.

4 Conclusions

This deliverable is the first version of the data management plan of UNITI and has described how data collected for the purposes of the project will be handled during and after the end of the project.

In summary, UNITI commits the specific security and privacy control measures to ensure the preservation of the integrity, availability and confidentiality of the data that will be collected in the project as well as the preservation of the regulatory requirements of the General Data Protection Directive (GDPR). The project also commits to making the data about its publications and the collected clinical data available as open source, if that will be allowed by ethics and other requirements of the clinical partners who own the datasets.

As a final concluding remark, it should be noted that although, we have conducted an initial analysis aimed at envisaging what would be necessary for the above purposes. a possibility to encounter a need to revise it. If such a need arises during of the project, the data management plan will be amended and communicated to all stakeholders, who should be notified about the amendments.

---

2 https://data.europa.eu/euodp/en/data/dataset/open-research-data-the-uptake-of-the-pilot-in-the-first-calls-of-horizon-2020

3 https://data.europa.eu/euodp/en/home
