# Supplementary material for: Unification of Treatments and Interventions for Tinnitus Patients (UNITI): a study protocol for a multi-center randomized clinical trial
Source: Trials. 2021 Dec 4;22:875. doi: 10.1186/s13063-021-05835-z (PMC8642746; doi:10.1186/s13063-021-05835-z)
Supplement: Supplementary file 1 — Additional file 1. Ethical approvals from Germany, Spain, Greece and Belgium. Informed consent form – RCT. Information sheet – RCT. Informed consent form – blood sampling. Information sheet – blood sampling. UNITI data management plan. WHO trial registration dataset. [file 13063_2021_5835_MOESM1_ESM.zip › UNITI_information_sheet_bloodsamplingR1.pdf]

INFORMED CONSENT FORM

Study Title: Unification of treatments and Interventions for Tinnitus patients – Genetic analysis

Study Code: UNITI-Gen

Study team: ……

Principal Investigator: …………………………………………………………………………………………

Site: ……

Contact details: …………………………………………………………………………………………………

Patient Code: ……………………………………………………………………………………………………

Dear study participant,

an optional, but essential part of this study involves the collection of a blood sample from you during the study which will be used to research the genetic component of tinnitus. Before you decide whether to participate in this part of the study we would like to give you some more information to help you to understand why the genetic research is being done, what is involved and how your data will be used. Together with a member of the study team, you will now go through this Informed Consent Form (ICF) which has two parts.

- **Information Sheet** – for information on this research project
- **Certificate of Consent** - for signatures if you agree to take part

If you have any questions or difficulties understanding the following information, please do not hesitate to ask questions.

You will be given a copy of the complete Informed Consent Form

PART 1: INFORMATION SHEET – Genetic Analysis

1. WHY DOES THIS GENETIC RESEARCH MATTER?

Additional genetic analyses for research purposes will be performed on blood samples taken once at treatment period while you are on the study.

Genetic research looks at genetic material, which is in your body, such as in blood cells. Cells are the ‘building blocks’ of your body. Cells contain a type of molecule called deoxyribonucleic acid (DNA). Your genes are made of DNA. There are other components of genetic material as well, such as RNA (ribonucleic acid). You can think of genetic

information as a large instruction book that your body reads to understand how it should be built and function.

All humans have the same instruction book in their body but some words or letters may be different from one person to the other. Some of those differences have no effect on your health, like hair or eye color; but others can influence the likelihood of developing a disease or affect how medicine to treat a certain disease will work. If genetic analyses are done, they may involve all or part of your genetic information. Some genetic information is inherited from your parents and some genetic information is only found in your body and cannot be passed on from generation to generation.

We ask you and other participants in this study to provide blood samples because we want to investigate how genetic differences between people and their disease can affect the way people respond to the treatment used in this study. In other words, we want to identify genetic factors that may be responsible for the occurrence/expression and severity of your tinnitus and for your response to a particular treatment

The blood samples collected are sent to specific laboratories (Granada, Spain; Stockholm, Sweden) for specific analysis (in accordance with EU data protection legislation). The genetic test will not bring you any direct personal benefit and the results will not be available to you, your family or your doctor and will not affect your treatment. We will only use your samples for the purpose set out in this document. Blood collection is completely voluntary for you

If you decide not to take a blood sample, this has no influence on your participation in the study.

If you withdraw your consent after taking a sample but before your blood sample is analysed, the study team will arrange for the sample to be destroyed.

If you withdraw your consent after your blood sample has been analysed, the study team/principal investigator will make all reasonable efforts to destroy your blood sample and any extracted DNA, and your data will not be included in any future analysis or research, but the study team/principal investigator (PI) is under no obligation to destroy the existing analysis of this study.

1. WHAT WILL HAPPEN IF I DECIDE TO TAKE PART IN THIS GENETIC RESEARCH?

If you would like to provide a blood sample for genetic research, we will take about 12 ml of blood from you at any time point during the study. DNA will be extracted from your sample. In this process, most of the original blood sample will be used, but a small amount may be kept as a “backup” in case there are of problems with the analysis of your DNA.

The DNA and the remaining sample will be sent from the respective study centres to the above-mentioned two laboratories in Spain and Sweden according to current regulations, where it will be stored and analysed under secure conditions.

DNA and/or the blood sample will be kept for up to 5 years after the main study is completed and then it will be destroyed.

The genetic analyses are carried out exclusively in the two laboratories mentioned above. These laboratories are obliged to comply with the agreements in this Informed Consent.

2. WHAT ARE THE POSSIBLE MEDICAL RISKS OF TAKING PART?

Blood collection poses only a minimal risk because all the material used is sterile. Nevertheless, in rare cases an infection can occur. Temporary pain at the injection site, a haematoma ("bruise") or reddening of the skin may also occur

3. HOW WILL I BENEFIT FROM TAKING PART?

You will have no direct benefit from this research. However, this research may contribute to the better understanding of tinnitus condition and its treatment. This genetic research may eventually lead to improvements in diagnosis and treatment.

4. DO I HAVE TO TAKE PART?

Your blood sample is voluntary. You will receive the same treatment and care in the study, regardless of whether you give blood of this genetic research or not. If you decide not to give blood, you can still participate in the study.

5. DO I RECEIVE A PAYMENT FOR TAKING PART?

You will not be paid for taking part in this research.

6. WILL I BE ABLE TO SEE MY RESULTS OF THE GENETIC RESEARCH?

This genetic study is for research purposes only. You will therefore not be able to view the results of your genetic testing.

7. DO I HAVE RIGHTS TO THE RESULTS FROM THE GENETIC RESEARCH?

You have no rights to the information from the genetic test. All information derived directly or indirectly from this genetic research, and all patents, diagnostic tests, pharmaceuticals or biological products developed directly or indirectly as a result of this genetic research are the sole property of the study team/principal investigator (PI) (and its successors, licensees and assigns) and may be used for commercial purposes. You have no right to this property or to any share of the profits that may be made directly or indirectly as a result of this genetic research. However, in signing this form and donating a blood sample for genetic research, you have the same rights as described in the Information Sheet for the main study.

8. HOW WILL MY GENETIC AND PERSONAL DATA BE USED AND PROTECTED?

This genetic research will be carried out with a high degree of confidentiality. Your blood sample will be labelled with the same code that is given to you in the study. No personal identifiers such as your name or date of birth will be recorded on the sample.

The results of this genetic research will not be linked to your identity. Personal identifying data (source data) will be stored separately from the data collected in the study in a secure location. In order to verify the proper conduct of the genetic research and the entire study, the responsible regulatory authorities may request access to your study documents including the original data (source data). The regulatory authorities can also verify that this genetic research has been carried out properly and can access your medical records and learn your identity for this purpose.

The set of your information collected in the study will be used together with the results of this genetic examination. This may include information about your tinnitus, your health, or your response to the treatment you received in the study. To keep this information confidential, personal information (such as name, address, social security number) will be removed and tracked using only a code number

The joint analysis of the genetic data and the other study data is necessary to achieve the objectives of this genetic study. The results of this genetic examination, such as the differences in your DNA compared to other people, will only be made available to the members of the study team conducting the genetic examination and will be kept under safe conditions at all times. Your sample will be analysed in the laboratory of the ENT Clinic Virgen de las Nieves and the Centre for Genomics and Oncological Research GENYO in Granada, Spain and in the Physiology and Pharmacology Laboratory of the Karolinska Institute in Stockholm, Sweden. All effluents are carefully monitored and no attempt is made to identify you from the available data.

In addition to this genetic examination and with the aim of improving science, patient care and public health, the study team/principal investigator (PI) and the organisations designated by him/her may carry out further research in the future, sharing summary results (e.g. genetic differences of groups of people with a disease) from this genetic examination with other researchers, such as hospitals, academic organisations or health insurance companies. The researchers exchange the results of this genetic analysis by posting them in scientific databases where they can be combined with the results of similar studies to learn more about health and disease. Researchers can use this information only for health-related research purposes. Researchers can see summary results that include your data, but they cannot see your individual data or information that identifies you personally. The results of this genetic research may also be published in scientific journals, but no information that identifies you personally will be included.

The study team/principal investigator (PI) will not disclose individual genetic data to persons who are not part of the study team.

<u>Specific information on data handling in the context of this study can be found in a separate document on data protection.</u>

9. CAN I WITHDRAW MY CONSENT?

You can withdraw your consent to use your sample in genetic research at any time.

If you withdraw your consent after taking a sample but before your blood sample is analysed, the study team will arrange for the sample to be destroyed.

If you withdraw your consent after your blood sample has been analysed, the study team/principal investigator will make all reasonable efforts to destroy your blood sample and any extracted DNA, and your data will not be included in any future analysis or research, but the study team/principal investigator (PI) is under no obligation to destroy the existing analysis of this study.

....	....
**Place Date**	**Place/Date**
....	....
**Participant´s name**	**Study team member´s name**
....	....
**Participant´s signature**	**Study team member´s signature**
