# Supplementary material for: Unification of Treatments and Interventions for Tinnitus Patients (UNITI): a study protocol for a multi-center randomized clinical trial
Source: Trials. 2021 Dec 4;22:875. doi: 10.1186/s13063-021-05835-z (PMC8642746; doi:10.1186/s13063-021-05835-z)
Supplement: Supplementary file 1 — Additional file 1. Ethical approvals from Germany, Spain, Greece and Belgium. Informed consent form – RCT. Information sheet – RCT. Informed consent form – blood sampling. Information sheet – blood sampling. UNITI data management plan. WHO trial registration dataset. [file 13063_2021_5835_MOESM1_ESM.zip › UNITI_information_sheet_RCTR1.pdf]

INFORMED CONSENT FORM

Study Title: Unification of treatments and Interventions for Tinnitus Patients – Randomized Clinical Trial

Study Code: UNITI-RCT

Study team (institution): ……………………………………………………………………………………………

Principal Investigator: ……………………………………………………………………………………………

Site (City): ………

Contact details: ……………………………………………………………………………………………………

Patient Code: ………………………………………………………………………………………………………

Dear study participant,

thank you very much for your interest in participating in this clinical trial, which will investigate different types of single and combined treatment types for tinnitus. Together with a member of the study team, you will now go through this Informed Consent Form (ICF) which has two parts.

- **Information Sheet** - for information on the progress of the study
- **Certificate of Consent** - for signatures if you agree to take part

If you have any questions or difficulties understanding the following information, please do not hesitate to ask questions.

You will receive a copy of the full Informed Consent Form

PART 1: INFORMATION SHEET - RCT

You are being asked to participate in this clinical research study. Before you decide whether to participate, it is important that you understand why this study is conducted, what is expected of you as a participant, the potential benefits, risks and inconveniences involved, and how your information and data will be used. Please read this information carefully and if you have any questions, please ask the study team. Before you make a decision regarding your participation, you are welcome to consult with others, such as your family doctor, family and friends. If you do not understand parts of the information presented to you, you are welcome to ask the study team to explain them to you. Should you have questions later, please do not hesitate to contact the study team. The contact details of the respective contact persons are listed at the end of this document.

This study is funded by the European Union´s Horizon 2020 research and innovation programme under the grant agreement no. 848261.

1. WHAT IS THE PURPOSE OF THE STUDY?

Tinnitus is the perception of noise or ringing without the presence of an external source of sound. It is a very common problem and many people are suffering from this condition. Although much progress has been made, there is currently no curative treatment for tinnitus available. Different manifestations of tinnitus make it difficult to find a treatment that benefits all those affected. However, in some people particular treatments appear to alleviate tinnitus-related suffering. The aim of this study is not only to compare commonly used tinnitus treatments (sound therapy, cognitive behavioural therapy, hearing aids or structured counseling), but also to investigate the effect of certain combinations of treatment approaches (e.g., hearing aids together with structured counseling). We further want to find out, who is likely to benefit from certain single- or combination treatments.

2. DO I HAVE TO TAKE PART?

Your participation in the study is entirely voluntary. You decide whether you want to participate or not. If you decide to participate, you are free to cancel your participation at any time and without giving reasons. However, if you decide to withdraw from the study, we ask you to seek advice from the study team or the responsible staff doctor and attend one of the early termination visits. This will in no way affect your medical care in the future.

The study team can also decide to take you off the study at any time. The reasons that might cause your treatment to be stopped include, but are not limited to:

- The study team decides that the study treatment is not helping you or that it is not safe for you to continue treatment
- You start a treatment with another therapy for tinnitus
- You are unable to comply with the study requirements
- You are positive for COVID-19 and the study team considers that your condition will jeopardize your health
- The study is stopped by the study site or by <<insert name of country regulatory body>> or another regulatory body
- The study is stopped or paused by the study team or the principal investigator due to new safety data from other participating subjects on the study

3. PROCEDURE OF THE STUDY OR WHAT WILL HAPPEN IF I DECIDE TO TAKE PART?

We want to compare frequently used tinnitus treatments and investigate the combined effect of certain treatments. We further want to find out, who is likely to benefit from certain single- or combination treatments. As a first step, participants will be divided into two groups based on their tinnitus distress level (measured by a standardized tinnitus questionnaire; THI). In a next step, we will divide participants with a low or a high tinnitus distress grade into two groups based on their level of hearing (measured by a clinical hearing test). If your degree of hearing loss shows a need for a hearing aid, you will be placed in a group for hearing aid indication, which does not necessarily mean that you will receive a hearing aid. Within this group, participants are then randomly (by chance) assigned to one of 10 treatments (hearing aids, sound therapy, structured counseling, cognitive behavioral therapy as a single treatment or as combinational treatment with a maximum of two simultaneous treatments). If the degree of your hearing loss shows that you have no indication for a hearing aid, you will be included in the group without hearing aid indication. Within this group, participants are then also randomly (by chance) assigned to one of 6 treatments (sound therapy, structured counseling, cognitive behavioral therapy as a single treatment or as combinational treatment with a maximum of two simultaneous treatments). The study team will be looking after you and the other participants very carefully during the study. If there is anything that concerns you or makes you feel uncomfortable about this study, please talk to the study team. Regardless of the treatment you will receive, the study involved 3 phases: (1) The screening period, (2) the treatment period and (3) the follow-up period as shown in **Figure 1**. There will be a minimum of 4 visits (+ one additional voluntary visit), each lasting about 2 hours. Exception: For CBT there will be 12 additional group sessions (visits).

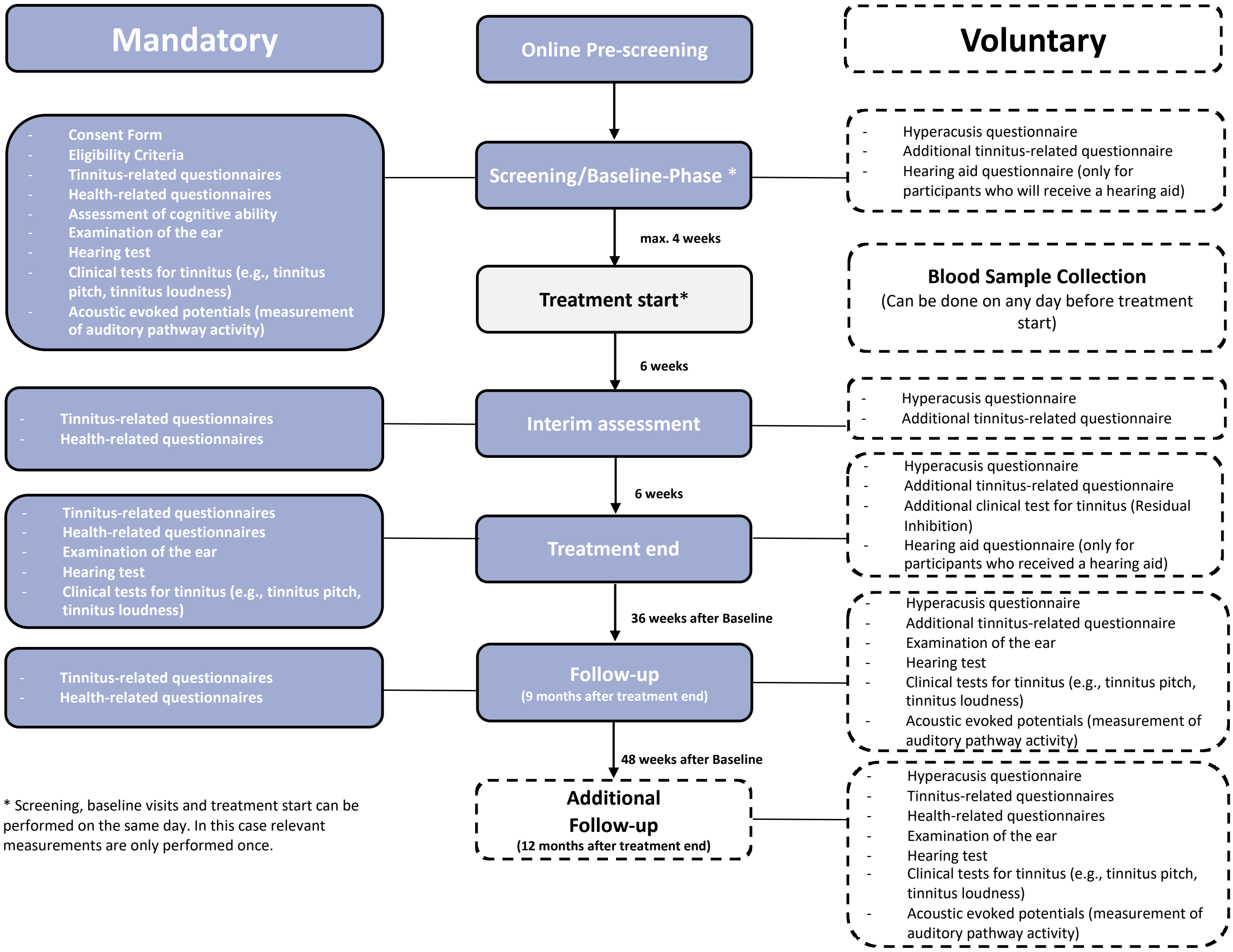

Figure 1 – Flow chart Assessments

Screening/ Baseline Period

Study procedures

In order to participate in this study, the screening period aims to clarify, if you are eligible for participation. The screening period is divided into an (1) online pre-screening and, if applicable, an (2) "on-site" screening.

After an affirmative (online) pre-screening, the study team will:

- ask you to sign the informed consent form.
- ask you to fill in some questionnaires and complete some tests that are relevant for study participation.
- will perform an assessment of your cognitive abilities.
- confirm that you are eligible for the study (or that you are not eligible).

If you are considered as eligible to participate, the study team and the responsible staff will:

- ask you to fill in some health-related questionnaires.
- evaluate your tinnitus with specific questionnaires.
- examine your ear.
- perform a hearing test.
- make some more clinical tests for tinnitus: tinnitus loudness, tinnitus pitch, tinnitus maskability, residual inhibition (short-term tinnitus suppression after sound stimulation).
- measure so-called acoustic evoked potentials (measurements of auditory pathway activity via electrodes placed on the head).
- record your full medical history, including all (a) prior treatments and medical problems you may have had and (b) treatments which are carried out during the course of the study.
- review any medication you are taking, including any over-the-counter medicines, herbal supplements, and herbal/natural remedies.
- ask you to give a small quantity of blood to measure so-called "biomarkers". A biomarker is a biological molecule that may or may not be related to the tinnitus characteristics mentioned above some biomarkers may help predict how well someone will respond to a specific treatment disorder. This part is optional. You do not have to give your consent to give blood for genetic analyses to take part in this study.
- randomize you to a treatment group.
- (If you are randomized to a hearing aid treatment group, you will be asked to fill out a short voluntary hearing aid questionnaire.)

All measures are usually completed within 4 weeks prior to the treatments starting. Should your treatment begin more than 4 weeks after your first screening assessment, the above mentioned measures will be obtained again (except for two specific questionnaires about your tinnitus, the measurement of acoustic evoked potentials and the examination of the ear).

Treatment Period

The treatment period will last 12 weeks. During the treatment period you will be asked to visit the clinic 3 times minimum.

Study procedures

Treatment Start: During the baseline visit you will be randomized to a specific treatment. The study team will

- explain your particular treatment to you in detail.
- update your medical history

Interim Visit: During the interim assessment (6 weeks after treatment start; approx. duration: 2h) the study team will

- ask you to fill in some health-related questionnaires.
- evaluate your tinnitus with specific questionnaires.
- update your medical history.
- ask you, if any adverse events occurred during the study period so far.

It is possible to complete the interim visit from home. For this purpose, you will be sent an e-mail with a specific URL-link for the completion of the questionnaires. Further, during a joint telephone call with a member of the study team, you will be asked questions about your medical history and any adverse events.

Final Visit (= Treatment End): During the Final Visit (12 weeks after treatment start; approx. duration: 2h), which is also the end of your treatment, the study team will:

- ask you to fill in some health-related questionnaires.
- evaluate your tinnitus with specific questionnaires.
- update your medical history.
- ask you, if any adverse events occurred during the study period.
- examine your ear.
- again perform a hearing test.
- make some more clinical tests for tinnitus: tinnitus loudness, tinnitus pitch, tinnitus maskability.
- ask you, if you want to participate in further voluntary clinical tests for tinnitus: residual inhibition (short-term tinnitus suppression after sound stimulation).

- (If you were in a hearing aid treatment group, you will be asked to fill out a short voluntary hearing aid questionnaire)

Follow-up Period

During the follow up period you are asked to visit the clinic 2 times as shown in **Figure 1**.

Study procedures

Follow up (approx. duration: 2h)**:** The 1st follow-up visit will be performed **9 months** after your baseline visit. Here, the study team will:

- ask you to fill in some health-related questionnaires.
- evaluate your tinnitus with specific questionnaires.
- update your medical history.
- ask you, if any adverse events occurred since the end of the treatment period.
- ask you, if you want to participate in further voluntary measurements:
  - examine your ear
  - again perform a hearing test
  - some more clinical tests for tinnitus: tinnitus loudness, tinnitus pitch, tinnitus maskability, residual Inhibition (short-term tinnitus suppression after sound stimulation).
  - measures of acoustic evoked potentials (measurements of auditory pathway activity via electrodes placed on your head).

Additional follow up (approx. duration: 2h)**:** The 2nd follow up visit will be performed 12 months after your baseline visit. This is a voluntary visit. During this visit the study team will:

- ask you to fill out some health-related questionnaires.
- evaluate your tinnitus with specific questionnaires.
- examine your ear.
- perform a hearing test.
- will make some more clinical tests for tinnitus: tinnitus loudness, tinnitus pitch, tinnitus maskability, residual Inhibition (short-term tinnitus suppression after sound stimulation).

- will perform measures of acoustic evoked potentials (measurements of auditory pathway activity via electrodes placed on your head).
- update your medical history.

ask you, if any adverse events occurred during the study period

Additional (to study-treatment and mandatory visits) voluntary assessment via mobile applications

The study team will ask you if you want to participate in an additional voluntary assessment. Herein, you have to answer several tinnitus-related questions via a specific app on your smartphone on a daily basis (5-10 minutes per day).

4. WHAT DO I HAVE TO DO?

If you agree to take part in this study, you must complete the tests and measures described above performed at the designated times before, during and after your treatment and come to the clinic at the times agreed with the study team. It is important that you follow all instructions from the study team or the responsible staff. In addition to the above, you must:

- provide accurate and complete information about your medical history and your present condition.
- inform the study team immediately if you believe you have symptoms of COVID-19.
- inform the study team about any new side effect, injury, or symptom you experience. Inform the study team about any changes in current medical conditions. This information should be reported to the study team between study visits by using the contact numbers on the first page of this information sheet.
- inform the study team about any other prescription or over-the-counter medication, vitamins, herbal supplements or herbal/natural remedies you are taking before and during the study. Check with the study team before starting any new medications or treatments.
- be able to complete the study:
    - i.e. have no plans for a sabbatical or long-term holiday
    - if you are a female – not be currently pregnant or plan to be in the near future. The treatment will not affect you or the baby, but please inform the study team about it.

5. WHAT ARE THE POSSIBLE SIDE EFFECTS, RISKS AND DISCOMFORTS OF TAKING PART?

- It is not expected that you will have more side effects than those you might experience from current clinical practice.
- As with any tinnitus investigation there is a possibility of a slight increase of tinnitus since you will pay more attention to your tinnitus. If you experience a severe long-term deterioration of your tinnitus, please inform the study team and do not quit the treatment by yourself.
- During the study, you will be asked to give a small quantity of blood. The procedure is carried out in a routine manner, however, in the extremely unlikely event of adverse reactions, the study team will follow you closely and keep track of any unwanted effects or problems. Blood collection poses only a minimal risk because all the material used is sterile. Nevertheless, in rare cases an infection can occur. Temporary pain at the injection site, a haematoma ("bruise") or reddening of the skin may also occur.
- In order to get as much information about you and your tinnitus as possible, you will be asked to complete many different questionnaires and measures will be conducted. This can sometimes be very time-consuming and demanding. But we are well aware of this and trying to find a good balance for any participant.

6. HOW WILL I BENEFIT FROM TAKING PART?

It is hoped that the new treatment will help you with your tinnitus suffering, but this cannot be guaranteed and there may not be any direct benefit for you. Information from this study may help researchers understand tinnitus and develop new tests and treatments to help other patients with this condition.

7. WHAT IF NEW INFORMATION BECOMES AVAILABLE?

If important new information on the study treatment becomes available which may affect your decision to be in the study, the study team will promptly tell you. As a result of such new information, the study team might recommend that you leave the study. He/she will explain the reasons for this and will talk to you about how best to manage your condition.

8. WHAT ARE THE COSTS OF TAKING PART?

There are no costs for you if you take part. You will receive the treatment at no cost and there will be no charges for study visits, tests or procedures. You will not be paid for being in this study. If you are randomized to the group that will be given a hearing aid you are allowed to keep this device after the completion of the study.

9. HOW WILL MY PERSONAL DATA BE USED AND PROTECTED?

Your personal data is protected by all applicable data protection and privacy laws, which include EU Regulation 2016/679.

To get the answers we need from the research described in this document, we have to collect personal information about you and your health. This includes information collected at your clinic during this study and also information that is already in your medical records. Besides information about your health we need information such as your age and gender. Results from the tests and examinations mentioned in the section “Procedure of the study or what will happen if I decide to take part ” are also included as part of your personal information. The collected data will then be uploaded in an international tinnitus database for collaborating researchers and stored according to EU data security and privacy settings. No personalized data will be stored in the database.

All collected data will be treated strictly confidential within the legal framework. All information concerning your personal data is made anonymous, only the local study team at the study site will know who you are. Your name will be replaced by an Identifier Code and only selected members of the study team have access to this code. Health authorities and people helping the study team/ principal investigator to run the study, including members of ZEINCRO group will be allowed to see your personal information, but they will not know who you are unless they are study inspectors.

The study information from this study must be kept for at least 10 years (§ 13 GCP-V). The results will be used to learn about tinnitus and different treatment methods. They can also be used to answer other questions including the safety of treatment. The use of your coded personal data for these purposes is based on your consent, on legal requirements concerning the performance of research studies and on public interest.

The results of this study will be published, for example, in medical journals or online, but you will not be mentioned in a way that would allow the public to find out who you are. Researchers, such as those from other companies and universities, may ask to use information from this study, including your information and samples, for other medical, health or scientific research. Researchers may combine the results from this study with results from other studies. Any disclosure of study data for scientific purposes will only be made in an anonymous form, i.e. no conclusions can be drawn about you as an individual, and in accordance with this document. You have the right to object to the use of your data for this additional research for reasons specific to you. If you wish to object to such use, please contact the study team.

Your coded study data information will not be sent outside the European Economic Area (EEA).

You may request to see the information collected about you. If you believe that any of this information is incorrect, you can write to the study team to ask them to change or remove the incorrect information. You can also request that we may limit the use of your personal information. If you change your mind about participating, we will not be able to remove the personal information that was collected for this study before you stopped participating. If you have questions about how we use your personal information or if you would like a copy of the Binding Corporate Rules, please contact the study team first. You can also contact the responsible data protection officer:

Last Name/ First Name: <<insert name of country-specific name >>

Contact details:

If you are not satisfied with the answers you receive, or in the event of violations, you have the right to complain to the relevant supervisory authority

<<insert name of country-specific complaint office>>

The blood samples that you may provide will be used for specific analyses during this study. You will not receive copies of the results. If you decide to stop participating in this study, your study-coded information and samples we have already collected will continue to be used in the manner you agreed to at the beginning of the study.

Your samples are analyzed in another country (Spain, Sweden). The coded samples are stored there under secure conditions.

In the event of a breach of the use of your personal data, you will be notified immediately in accordance with the applicable legislation of such a breach.

With respect to the handling of your personal data we refer to the information sheet for data protection, which will be discussed with you separately.

10. BIOLOGICAL SAMPLES

This study involves the collection of biological samples such as blood. Blood will be collected for genetical analysis. For example, to identify a potential biomarker for tinnitus/ response to a certain treatment. Regardless, if you do agree or not agree to give blood for the above-mentioned analyses, you will be able to participate in this study.

If you are interested in giving a blood sample, please inform the study team member or the responsible staff so they can give you the specific ICF related to blood sampling.

11. WHAT OTHER INFORMATION WILL BE AVAILABLE?

A description of this clinical trial will be available on clinicaltrials.gov as required by National and International Law and no personal data will be public.

12. WHOM SHOULD I CONTACT IF I NEED MORE INFORMATION OR HELP?

If you have any questions or need clarifications for this study or in case of any side effects, feel free to contact the principal investigator whose contact details are on the first page of this document.

You have the right to ask questions at any time about the potential risks of this study. **Your participation in this study is voluntary and can be revoked at any time during the study period without giving reasons and without serious disadvantages for you.**

………………………………………	………………………………………
**Place Date**	**Place/Date**
…………………………………………………………	…………………………………………………………
**Participant´s name**	**Study team member´s name**
…………………………………………………………	…………………………………………………………
**Participant´s signature**	**Study team member´s signature**
