# Supplementary material for: Unification of Treatments and Interventions for Tinnitus Patients (UNITI): a study protocol for a multi-center randomized clinical trial
Source: Trials. 2021 Dec 4;22:875. doi: 10.1186/s13063-021-05835-z (PMC8642746; doi:10.1186/s13063-021-05835-z)
Supplement: Supplementary file 1 — Additional file 1. Ethical approvals from Germany, Spain, Greece and Belgium. Informed consent form – RCT. Information sheet – RCT. Informed consent form – blood sampling. Information sheet – blood sampling. UNITI data management plan. WHO trial registration dataset. [file 13063_2021_5835_MOESM1_ESM.zip › WHO_trials_registration_dataset_rev1R1.docx]

| **UNITI trial registration – data set** | |
| --- | --- |
| **Data category** | **Information** |
| **Primary registry and trial identifying number** | NCT04663828 |
| **Date of registration in primary registry** | 11. December 2020 |
| **Secondary identifying numbers** | N/A |
| **Source(s) of monetary or material support** | The UNITI project has received funding from the European Union’s Horizon 2020 Research and Innovation Program (grant agreement number 848261). |
| **Primary sponsor** | UNITI Consortium  UNITI-RCT clinical sites:   1. Katholieke Universiteit Leuven, Leuven, Belgium 2. Charité – Universitaetsmedizin Berlin, Berlin, Germany 3. Ethniko Kai Kapodistriako Panepistimo Athinon, Athens, Greece 4. Hospital Universitario Virgen de las Nieves/Hospital Clinico Universitario San Cecilio, Granada, Spain 5. Klinikum der Universitaet Regensburg, Regensburg, Germany |
| **Secondary sponsor(s)** | N/A |
| **Contact for public queries** | Winfried Schlee  Email: [winfried.schlee@gmail.com](mailto:winfried.schlee@gmail.com)  Stefan Schoisswohl  Email: Stefan.schoisswohl@ukr.de |
| **Contact for scientific queries** | Winfried Schlee  Email: [winfried.schlee@gmail.com](mailto:winfried.schlee@gmail.com)  Stefan Schoisswohl  Email: Stefan.schoisswohl@ukr.de |
| **Public title** | UNification of Treatments and Interventions for TInnitus Patients - Randomized Clinical Trial (UNITI-RCT) |
| **Scientific title** | UNification of Treatments and Interventions for TInnitus Patients - Randomized Clinical Trial (UNITI-RCT) |
| **Countries of recruitment** | Germany (Berlin, Regensburg), Greece (Athens), Spain (Granada), Belgium (Leuven) |
| **Health condition(s) or problem(s) studied** | Chronic tinnitus |
| **Intervention(s)** | Hearing Aids:  Hearing aids are considered medical devices and are recommended to patients with a certain degree of hearing loss. Hearing aids amplify environmental sounds.  Sound Therapy:  Several sounds and music are used for tinnitus disorder  Cognitive Behavioral Therapy:  This is a structured intervention and consists of a several week program both with noise exposure and structured interviews and expert-patient as well as patient-patient interaction in groups  Structured Counseling:  Structured patient education and counselling is considered as providing information to patients in order to empower them to better manage their disorder |
| **Key inclusion and exclusion criteria** | Inclusion Criteria:   - Primary complaint tinnitus - Chronic tinnitus (for at least 6 months based on history) - Age 18-80 years - Ability to understand and consent to the research / ability to participate (hearing ability, intellectual capacity, no plans for sabbaticals or long-term holidays, no (plans for) pregnancy*) - A score of >22 on the Montreal Cognitive Assessment (MoCa), i.e. adults without mild cognitive impairment - Ability and willingness to use the UNITI mobile applications on their smartphones - A score of ≥ 18 in the Tinnitus Handicap Inventory (THI) of Newman et al. (1996) - Willing to use a hearing aid (if indication) - If a drug therapy with psychoactive substances (e.g. antidepressants, anticonvulsants) exists at the beginning of the therapeutic intervention, it must have been stable for at least 30 days. The therapy should remain constant during the duration of the study, but a necessary change is not an exclusion criterion. Any change in medication is documented in the CRF.   Exclusion Criteria:   - Objective tinnitus / heartbeat-synchronous tinnitus as primary complaint - Start of any other tinnitus related treatments, especially hearing aids, structured counseling, sound therapy (with special devices; expecting long term effects) or cognitive behavioral therapy in the last 3 months before the start of the study** - Otosclerosis / acoustic neuroma or other relevant ear disorders with fluctuation hearing - Present acute infections (acute otitis media, otitis externa, acute sinusitis) - Meniere's disease or similar syndromes (but not vestibular migraine) - Serious internal, neurological or psychiatric conditions - Epilepsy or other CNS disorders (brain tumor, encephalitis) - Clinically relevant drug, medication or alcohol abuse up to 12 weeks before study start - Missing written informed consent - Severe hearing loss – inability to communicate properly in the course of the study; evaluated by examiner - One deaf ear   *Due to specific standards of the local ethics committee at the clinical site in Granada, Spain (Servicio Andaluz de Salud) with respect to the conduction of RCTs, all female participants will be tested with regards to an existing pregnancy.  ** If a HA has already been worn three months before screening, eligible candidates are allowed to participate, but are automatically assigned to the no HA indication group. |
| **Study type** | Interventional; multi-center, parallel-arm RCT |
| **Date of first enrolment** | Berlin, Germany - April 2021  Regensburg, Germany – April 2021  Granada, Spain – June 2021  Athens, Greece – June 2021 |
| **Target sample size** | 100 per clinical center; in total 500 participants |
| **Recruitment status** | Recruitment is ongoing (pre-screening & screening) |
| **Primary outcome(s)** | The primary outcome will cover the domain tinnitus distress. The total score in the Tinnitus Handicap Inventory (THI; Newman et al., (1996)) is defined as the primary outcome measure for tinnitus distress. |
| **Key secondary outcomes** | Several other standardized tinnitus- and health-related questionnaires will be used as secondary outcomes in the course of this RCT:   1. Tinnitus Functional Index (THI; Meikle et al., 2011) 2. Mini Tinnitus Questionnaire (mini-TQ; Hiller and Goebel, 2004) 3. Tinnitus numeric rating scales (NRS; Landgrebe et al., 2010) 4. World Health Organization – Quality of Life abbreviated (WHOQoL-Bref; <https://www.who.int/healthinfo/survey/WHOQOL_BREF.pdf?ua=1>) 5. Clinical Global Impression Scale - Improvement (CGI-I; Adamchic et al., 2012) 6. Patient Health Questionnaire for Depression (PHQ-D/PHQ-9; Kroenke et al., 2001; Kroenke and Spitzer, 2002) |
| **Ethics review** | Status:   - Berlin & Regensburg – positive ethics votum (22. July 2020) - Granada – positive ethics votum (28. January 2021) - Athens – positive ethics votum (26. of January 2021) - Leuven – positive ethics votum (15. of October 2021) |
| **Completion date** | End of 2022 (final data collection date for primary outcome) |
| **Summary Results** | The results of the study will be submitted for publication in peer reviewed journals without the possibility of conclusions regarding participants personal data |
| **IPD sharing statement** | Data will be collected at the clinical sites and entered in the UNITI database. Data will be treated as securely as possible and protected according to the latest international guidelines. In terms of data protection personal data will be managed according to EU guidelines. All personal data will be treated with the upmost confidentiality and multiple efforts will be made in order to anonymize or pseudo-anonymize the collected data and to protect participants’ identities. |
